# In vivo screening reveals interactions between *Drosophila Manf* and genes involved in the mitochondria and the ubiquinone synthesis pathway

**DOI:** 10.1186/s12863-017-0509-3

**Published:** 2017-06-02

**Authors:** Riitta Lindström, Päivi Lindholm, Mari Palgi, Mart Saarma, Tapio I. Heino

**Affiliations:** 1 Department of Biosciences, University of Helsinki, FI-00014 Helsinki, Finland; 2 Institute of Biotechnology, University of Helsinki, FI-00014 Helsinki, Finland; 3 Department of Chemistry and Biotechnology, Tallinn University of Technology, EE-12618 Tallinn, Estonia; 4 Current affiliation: Institute of Biotechnology, University of Helsinki, Helsinki, Finland

**Keywords:** MANF, CDNF, Genetic screen, Mitochondria, Ubiquinone

## Abstract

**Background:**

Mesencephalic Astrocyte-derived Neurotrophic Factor (MANF) and Cerebral Dopamine Neurotrophic Factor (CDNF) form an evolutionarily conserved family of neurotrophic factors. Orthologues for MANF/CDNF are the only neurotrophic factors as yet identified in invertebrates with conserved amino acid sequence. Previous studies indicate that mammalian MANF and CDNF support and protect brain dopaminergic system in non-cell-autonomous manner. However, MANF has also been shown to function intracellularly in the endoplasmic reticulum. To date, the knowledge on the interacting partners of MANF/CDNF and signaling pathways they activate is rudimentary. Here, we have employed the *Drosophila* genetics to screen for potential interaction partners of *Drosophila Manf* (*DmManf*) in vivo.

**Results:**

We first show that DmManf plays a role in the development of *Drosophila* wing. We exploited this function by using *Drosophila* UAS-RNAi lines and discovered novel genetic interactions of *DmManf* with genes known to function in the mitochondria. We also found evidence of an interaction between *DmManf* and the *Drosophila* homologue encoding Ku70, the closest structural homologue of SAP domain of mammalian MANF.

**Conclusions:**

In addition to the previously known functions of MANF/CDNF protein family, *DmManf* also interacts with mitochondria-related genes. Our data supports the functional importance of these evolutionarily significant proteins and provides new insights for the future studies.

**Electronic supplementary material:**

The online version of this article (doi:10.1186/s12863-017-0509-3) contains supplementary material, which is available to authorized users.

## Background

An evolutionarily conserved protein family, MANF/CDNF family, is the most recently discovered family of neurotrophic factors (NTFs) [1–4]. Typically of NTFs, MANF and CDNF are small secreted molecules that support the survival of neurons [1, 2]. Mammalian MANF and CDNF support the brain dopaminergic system in rodent models of Parkinson’s disease (PD) in vivo [2, 5, 6]. MANF has been shown to protect neurons and cardiomyocytes against ischemic injury in extracellular manner [7, 8]. Additionally, MANF is required for the proliferation and survival of the pancreatic β-cells [9].

Orthologues for MANF/CDNF are the only neurotrophic factors as yet identified in invertebrates with conserved amino acid sequence [1, 3]. The invertebrate homologues show higher similarity to mammalian MANF than CDNF [2, 3]. However, both human MANF and CDNF are functional orthologues of *Drosophila Manf* (*DmManf*) [3, 10]. In *Drosophila*, glial-derived DmManf is necessary for maintaining the neurites of embryonic and larval dopaminergic neurons that do not express DmManf. This demonstrates that the extracellular trophic function for dopaminergic system is conserved [3].

The knowledge on the molecular interactions of MANF/CDNF family proteins remains limited. Also the receptor for MANF/CDNF proteins is not known. Intracellularly, mammalian MANF has been shown to bind GRP78/BiP (Glucose-regulated protein 78/Binding immunoglobulin protein), the major ER chaperone, in Ca^2+^-dependent manner [8]. There is also experimental evidence suggesting that MANF interacts with KDEL-Rs, KDEL (Lys-Asp-Glu-Leu) endoplasmic reticulum protein retention receptors [11]. Furthermore, a recent study suggests that MANF interacts with a member of ER-associated reticulon protein family [12]. Our previous study shows a genetic interaction between *DmManf* and *Drosophila* homologues of *GRP78*, *PERK* (PRKR-like endoplasmic reticulum kinase, one of the ER stress sensor proteins) and *Xbp1* (X-box Binding Protein-1, a transcription factor mainly mediating ER stress response activated gene expression) [13]. Additionally, our earlier microarray analysis suggests that DmManf has a role in *Drosophila* ER stress response [14]. MANF is localized to ER [14–17] and the retention is mediated through the non-classical but evolutionarily conserved ER retention signal sequence, RTDL in human and RSEL in *Drosophila* [8, 10, 17]. Furthermore, the expression of *Manf* mRNA is induced in response to ER stress [13, 15, 17–20]. In addition to GRP78, co-immunoprecipitation studies have revealed that MANF (also known as Armet) interacts with a mutant form of an extracellular matrix protein matrilin 3 [21].

Both mammalian and *Drosophila* MANF have been shown to hold intracellular cytoprotective function against Bax (BCL-2 associated X) -dependent cell death in vitro [10, 22]. The C-terminal domain of MANF shows high structural homology to SAP (SAF-A/B, Acinus and PIAS) domain of Ku70 (Ku autoantigen p70 subunit), an inhibitor of Bax-mediated apoptosis [23], and it is alone capable of protecting neurons from induced apoptosis in vitro [10, 22].

MANF and CDNF have been suggested to be involved in inflammatory responses [24–28]. The main mediator of proinflammatory response, NF-κB (nuclear factor kappa-light-chain-enhancer of activated B cells), is also regulated by unfolded protein response, a cellular process activated by ER stress (reviewed e.g. in [29]). In a recent study MANF was found to bind the p65 subunit of NF-κB via the C-terminal SAP-domain in vitro [28]. Upon inflammation, MANF localized to nucleus and was suggested to suppress the expression of NF-κB targets by binding to DNA binding domain of p65 as well as to adjacent enhancer regions of target genes [28]. Interestingly, recent study demonstrated that MANF has a conserved immune modulatory function in both *Drosophila* and mouse promoting tissue repair and regeneration in retina [30].

In this work we used RNA interference (RNAi) approach in UAS/GAL4 in vivo system to study interacting partners of *DmManf* in *Drosophila* model. In the binary UAS/GAL4 system, GAL4 lines with various expression patterns are used for tissue-specific expression of UAS (upstream activation sequence) -transgenes [31]. RNAi where double stranded RNA (dsRNA) induces the degradation of targeted mRNA [32] is commonly used for gene silencing. Transgenic genome-wide *Drosophila* RNAi libraries have been established [33] (http://www.shigen.nig.ac.jp/fly/nigfly/) by introducing dsRNAs under UAS promotor. Crossing these flies with different GAL4 driver lines enables tissue-specific target gene inactivation. Expression of other UAS constructs or markers (e.g. GFP) can be simultaneously activated in the same GAL4 expression pattern. In this study, we used UAS-*DmManf*-RNAi construct for targeted knockdown of *DmManf* and performed a partial, unbiased screen of RNAi libraries in vivo to discover novel interacting partners for *DmManf*. Here we demonstrate genetic interactions between *DmManf* and genes with mitochondrial function.

## Results

### Silencing of *DmManf* by UAS-*DmManf*-RNAi is effective and specific in vivo

Homozygous *DmManf* mutants die at early developmental stage [3]. To study the role of DmManf during later stages of development we used the UAS/GAL4 system for tissue-specific knockdown of *DmManf* [31, 33]. Three UAS-*DmManf*-RNAi fly stocks were obtained from Vienna *Drosophila* RNAi Center (VDRC) (A in Additional file 1). All transformant lines showed similar phenotypes with different GAL4 drivers (B in Additional file 1), and the transformant line 12835 with construct ID 4793 was used in further experiments.

**Fig. 1:**
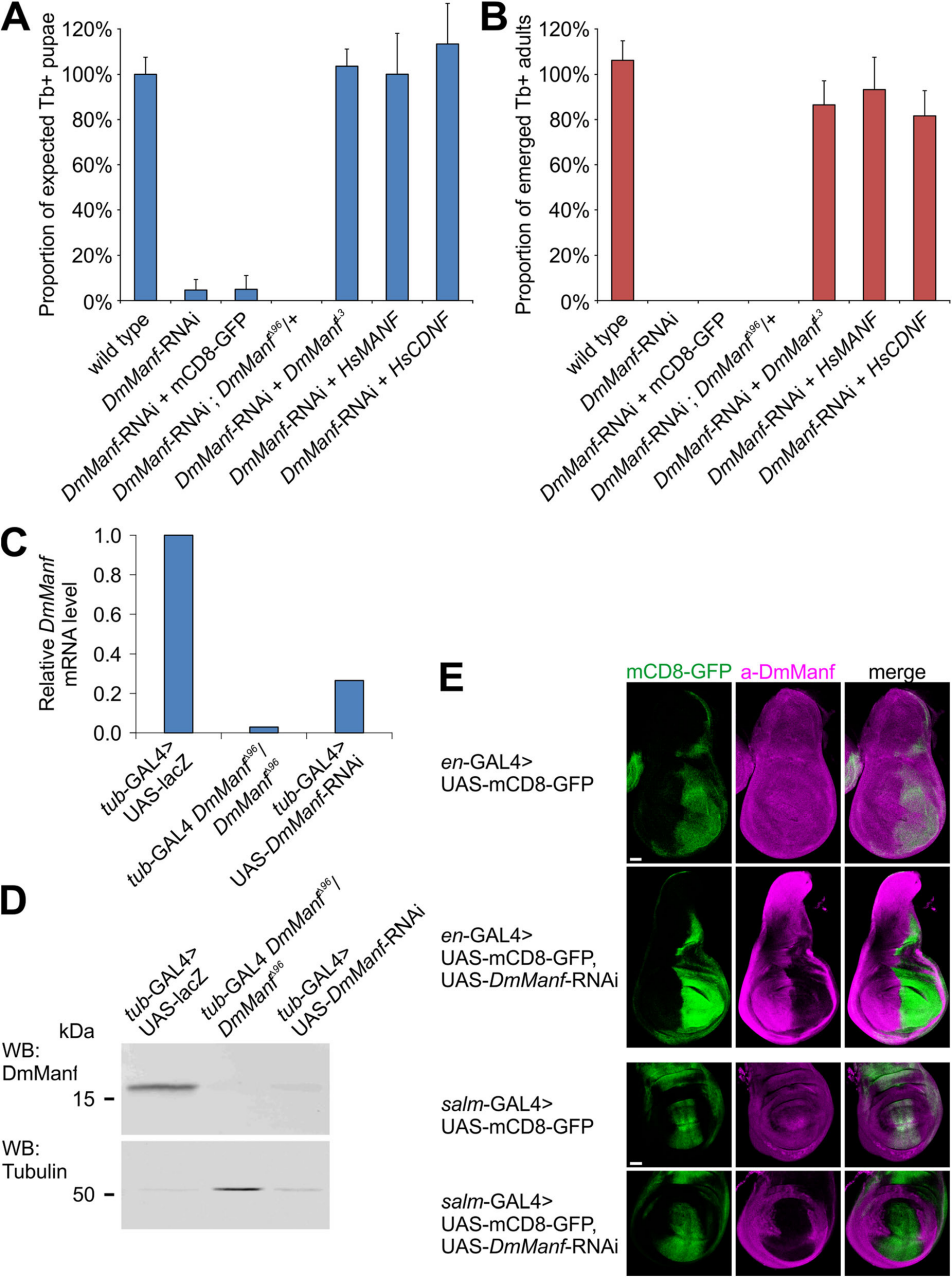
Knockdown of *DmManf* by UAS-*DmManf*-RNAi is effective and specific. **a**-**b** Quantification of pupae (**a**) and adults (**b**) of ubiquitous knockdown of *DmManf* with *tub*-GAL4 driver. Ubiquitous knockdown of *DmManf* was lethal at larval stage with few escapers to pupal stage. UAS-mCD8-GFP was used as a dose control for UAS/GAL4 binary expression system, *tub*-GAL4/+ as a wild type control. Amount of pupae and adults analysed are presented in Additional file 8. Proportion of Tb^+^ pupae was normalized to experimentally determined proportion of Tb^+^ pupae (see Additional file 8, wild type and wild type/SM6-TM6). **c**-**d** Quantitative RT-PCR (**c**) and Western blot (**d**) analyses revealed that ubiquitous knockdown of *DmManf* resulted in robustly reduced *DmManf* mRNA and protein levels in larvae collected 50–54 h after egg laying. Homozygous *DmManf* ^*Δ96*^ mutant showed diminished level of maternally contributed *DmManf* mRNA and protein. *tub*-GAL4 > UAS-*lacZ* flies were used as a wild type control. *DmManf* mRNA levels were normalized to wild type. Alpha-tubulin was used as a loading control for Western blot analysis. **e** Knockdown of *DmManf* with *en*-GAL4 and *salm*-GAL4 drivers showed a loss of DmManf immunoreactivity (*magenta*) in GAL4-expressing pattern. UAS-mCD8-GFP was used to detect GAL4 expression (*green*). Single laser confocal sections. Scale bar 50 μm

The ubiquitous knockdown of *DmManf* with *tub*-GAL4 and *da*-GAL4 drivers in wild type background showed lethal phenotype at larval to pupal stage (Fig. 1a–b and data not shown). The knockdown efficiency of *DmManf* expression was verified at both mRNA and protein level by quantitative RT-PCR (qPCR) and Western blot analyses, respectively (Fig. 1c–d). When the ubiquitous knockdown of *DmManf* was performed in heterozygous *DmManf* ^*Δ96*^ mutant background with already decreased DmManf protein level, the lethality was observed at early larval stage (Fig. 1a) resembling the phenotype of homozygous *DmManf* ^*Δ96*^ mutants [3].

According to information provided by VDRC, there are no predicted off-targets for *DmManf*-RNAi construct ID 4793. To verify the specificity of *DmManf*-RNAi, *DmManf* was simultaneously overexpressed (by UAS-*DmManf* ^L3^) and knocked down (by UAS-*DmManf*-RNAi) with ubiquitous *tub*-GAL4 driver. The overexpression of *DmManf* rescued the pupal lethality phenotype of ubiquitous *DmManf* knockdown flies into adulthood (Fig. 1a–b). We also used overexpression of the UAS constructs encoding transcripts for *DmManf* human (Hs) orthologues, *HsMANF* and *HsCDNF*, which share less homology with the *DmManf*-RNAi construct (C in Additional file 1) than *DmManf*. Both *HsMANF* and *HsCDNF* rescued the pupal lethality observed in *DmManf* ubiquitous knockdown flies (Fig. 1a–b). When two UAS constructs are used in the same fly, GAL4 protein supply is shared by the two promotor regions and might lead to decreased expression of UAS targets. In the case of UAS-RNAi lines, this dose effect could compromise the knockdown efficiency. To confirm that the rescue of *DmManf* knockdown phenotype was not due to inefficient knockdown of *DmManf*, we used UAS-*DmManf*-RNAi; UAS-mCD8-GFP line as a dose control for UAS/GAL4 binary expression system. Ubiquitous knockdown of *DmManf* by UAS-*DmManf*-RNAi; UAS-mCD8-GFP with *tub*-GAL4 showed similar proportion of expected pupae to UAS-*DmManf*-RNAi alone (Fig. 1a–b).

### Wing-specific knockdown of *DmManf* drastically alters wing morphology and increases cell proliferation

DmManf is ubiquitously expressed in 3rd instar larval wing disc [10]. To verify the efficiency of tissue-specific silencing of *DmManf*, we used *salm*-GAL4 and *en*-GAL4 to knock down *DmManf* and simultaneously express UAS-mCD8-GFP to visualize the GAL4 expression pattern in the larval wing disc. The loss of DmManf immunoreactivity was detected exactly according to *salm*-GAL4 and *en*-GAL4 expression pattern in the wing disc (Fig. 1e) further demonstrating that the knockdown of *DmManf* was efficient at protein level. Interestingly, in *DmManf* knockdown wing discs we detected mild but clear increase of DmManf immunoreactivity in regions next to the GAL4 expressing area (Fig. 1e). This might indicate a compensatory regulation of DmManf expression in response to the partial loss of DmManf in the wing disc.

**Fig. 2:**
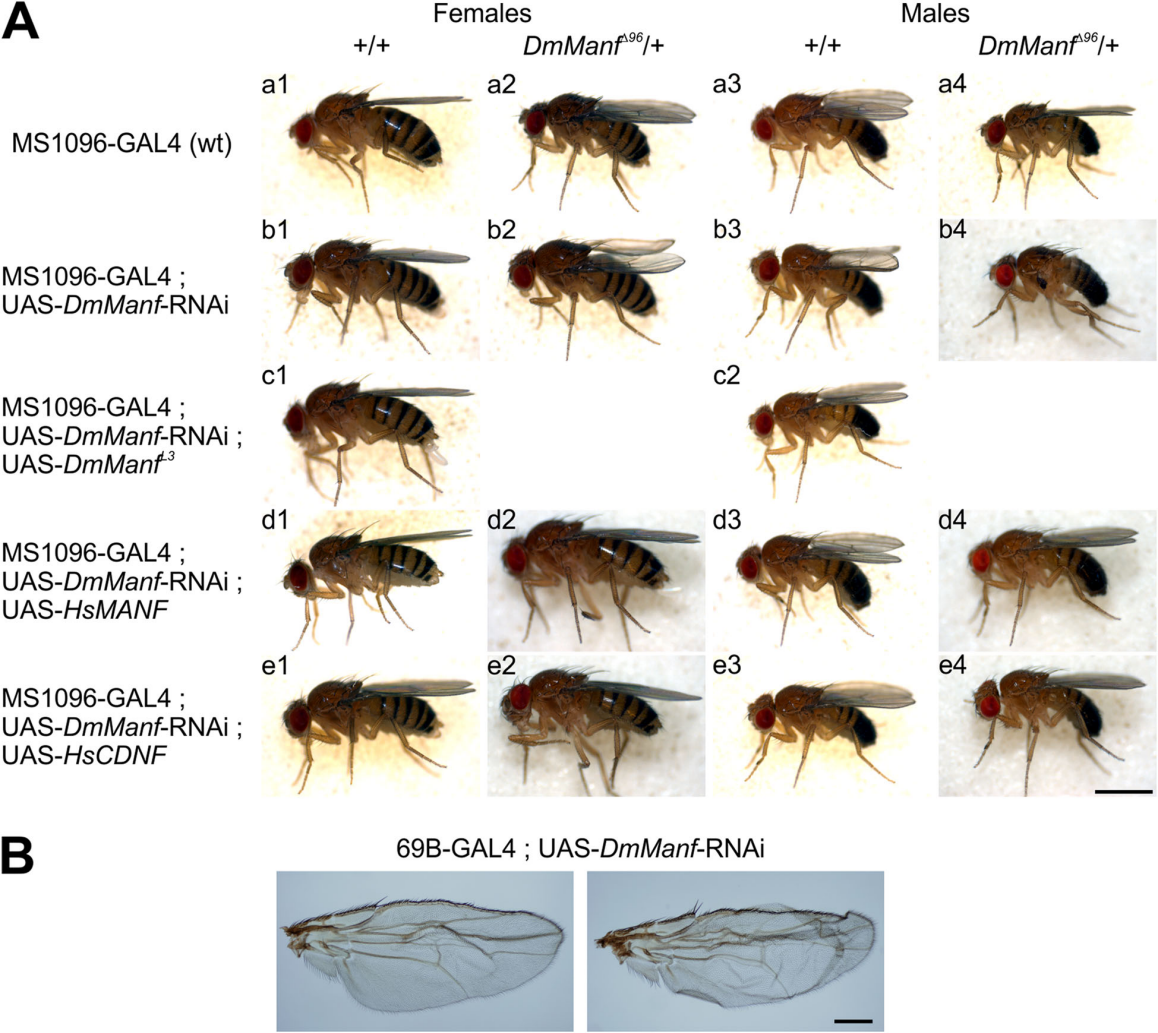
Loss of *DmManf* alters wing morphology. **a** Knockdown of *DmManf* with MS1096-GAL4 caused a “*bent-up*” phenotype in which the edges of wings were *curled upwards* (b3). *DmManf* knockdown in heterozygous *DmManf* ^*Δ96*^ mutant background showed more severe wing phenotype, even total loss of the wing blade in adult males (b2 and b4). Wing phenotype was rescued by overexpression of *DmManf* (c1-c2), *HsMANF* (d1-d4) and *HsCDNF* (e1-e4). MS1096-GAL4 flies were used as a wild type control (a1-a4). Scale bar 1 mm. **b** Knockdown of *DmManf* with semi-ubiquitous driver 69B-GAL4 showed a *blistered* wing phenotype in adult females. Males showed lethal phenotype in wild type background, females in heterozygous *DmManf* ^*Δ96*^ mutant background. Scale bar 100 μm

Interestingly, we observed two different wing phenotypes when various drivers with GAL4 expression in the wing were used to knock down *DmManf* in wild type background. The knockdown with MS1096-GAL4, A9-GAL4 and *Ser*-GAL4 drivers showed a three-dimensional “bent-up” wing phenotype (b3 in Fig. 2a and data not shown) while knockdown with 69B-GAL4 revealed blistered wing phenotype (Fig. 2b). The wing phenotype was observed with full penetrance in males of *DmManf* knockdown with MS1096-GAL4 (Table 1). To verify further the specificity of *DmManf* knockdown, we repeated the experiments in heterozygous *DmManf* ^*Δ96*^ mutant background. With 69B-GAL4, the knockdown of *DmManf* in heterozygous *DmManf* ^*Δ96*^ mutant background resulted in lethality. When MS1096-GAL4 driver was used to knock down *DmManf* in heterozygous *DmManf* ^*Δ96*^ mutant background, the wing phenotype was stronger as compared to *DmManf* knockdown in wild type background, especially in males in which the wing blade was totally lost (b2 and b4 in Fig. 2a). In comparison to females, the more severe knockdown wing phenotype observed in males was likely due to the dosage compensation of X-chromosomal GAL4 insertion in MS1096-GAL4 driver line.

**Table 1:** Penetrance of the wing phenotype in adult flies of *DmManf* knockdown with MS1096-GAL4

Genotype	Proportion of males with phenotype	Proportion of females with phenotype
MS1096-GAL4; UAS-*DmManf*-RNAi	140/140	102/176
MS1096-GAL4; UAS-mCD8-GFP	0/220	0/218

Simultaneous expression of UAS-*DmManf* ^L3^ and UAS-*DmManf*-RNAi with MS1096-GAL4 returned the wings back to wild type (c1-c2 in Fig. 2a) indicating that overexpression of DmManf could rescue the wing phenotype. Co-expression of UAS-*HsMANF* or UAS-*HsCDNF* together with UAS-*DmManf*-RNAi by MS1096-GAL4 also rescued the wing phenotype (d1-d4 and e1-e4 in Fig. 2a). This further demonstrated that the knockdown of *DmManf* by UAS-*DmManf*-RNAi construct was specific to the *DmManf* mRNA only and suggests that DmManf plays an important role during wing development.

**Fig. 3:**
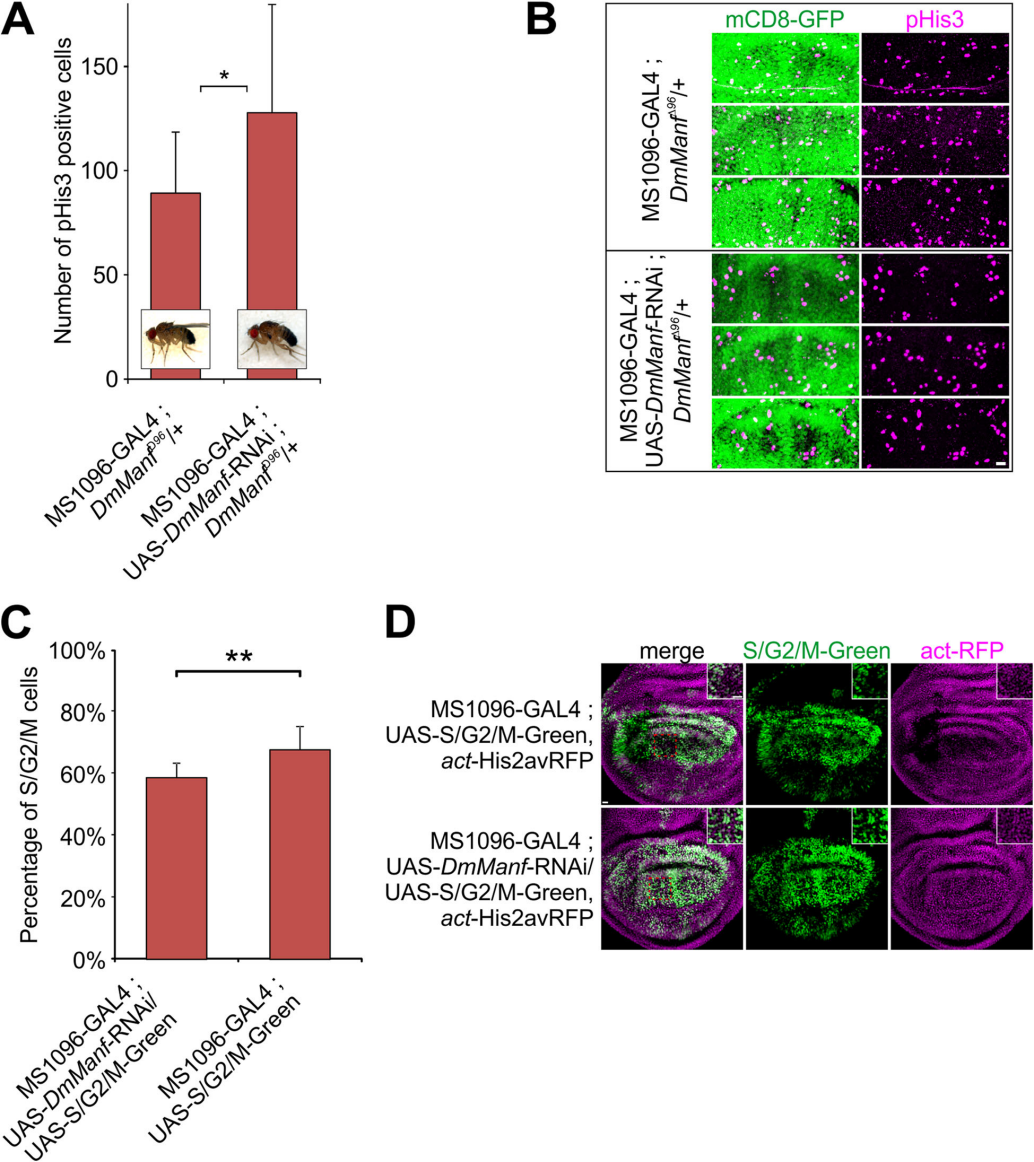
Knockdown of *DmManf* increases the number of mitotic cells in the wing disc. **a** Number of phosphorylated Histone-3 (pHis3) positive cells was significantly increased in GFP-expressing area of wing discs of third instar male larvae when *DmManf* was knocked down with MS1096-GAL4 in heterozygous *DmManf* ^*Δ96*^ mutant background. UAS-GFP.nls was used to detect GAL4 expressing area. *n* = 30. *, *P* < 0.05, Student’s t-test. **b** Three representative images of dorsal wing disc area showing pHis3 positive cells (*magenta*) and MS1096-GAL4 expression with UAS-GFP.nls (*green*). Scale bar 10 μm. **c**-**d** Proportion of S/G2/M positive cells (*green*) was increased in MS1096-GAL4 expressing wing discs of third instar male larvae in *DmManf* knockdown in comparison to wild type. *act*-His2Av-RFP (*magenta*) was used to mark all nuclei. Representative images of UAS-S/G2/M-Green driven with MS1096-GAL4 in wild type and together with UAS-*DmManf*-RNAi are shown (**d**). *Boxed* magnifications in *upper right corners* show the 37.9 μm × 37.9 μm area of dorsal wing disc compartment (*dashed rectangles in red*) used for analysis. Scale bar 10 μm. *n* = 11 for wt; *n* = 7 for *DmManf*-RNAi. **, *P* < 0.01, Student’s t-test

To investigate whether DmManf is involved in regulation of cell proliferation in vivo, we used the wing-specific knockdown of *DmManf* with MS1096-GAL4. Wing discs of third instar male larvae were stained for a mitotic marker phosphorylated Histone-3 (pHis3) [34]. We found that the number of pHis3 positive cells within GFP-expressing wing area was significantly increased when *DmManf* was knocked down in heterozygous *DmManf* ^*Δ96*^ mutant background in comparison to heterozygous *DmManf* ^*Δ96*^ mutant alone (Fig. 3a–b). In addition, we used UAS-S/G2/M-Green transgenic flies based on the Fucci (fluorescent, ubiquitination-based cell cycle indicator) model [35, 36]. *act*-His2AvRFP transgene was used to visualize nuclei of all cells. We fixed 3rd instar wing discs of male larvae and detected increased proportion of S/G2/M cells in *DmManf* knockdown with MS1096-GAL4 driver in comparison to wild type (Fig. 3c–d). This further indicates that the loss of DmManf affects cell cycle events.

### Screening of *DmManf* interaction partners

**Fig. 4:**
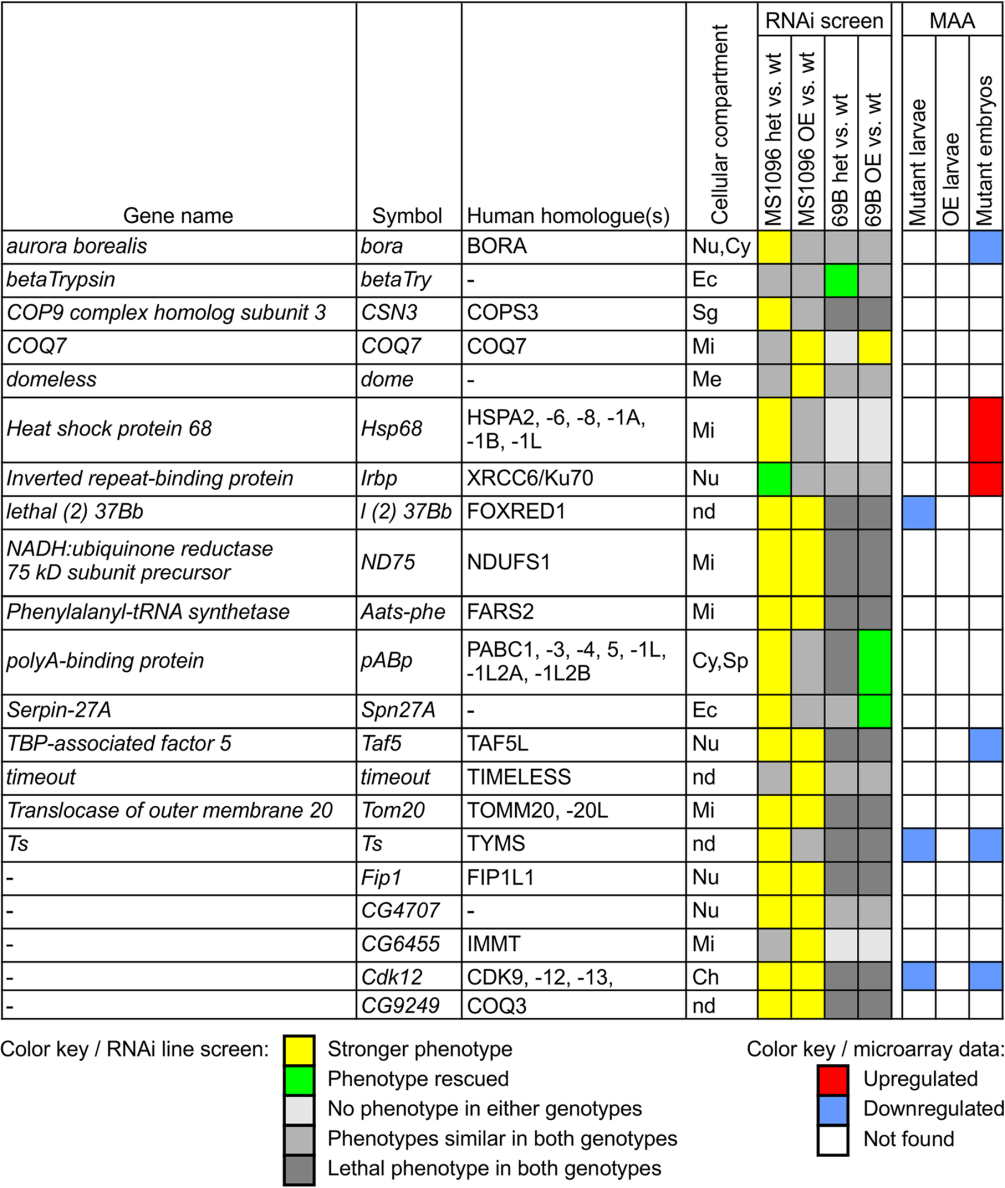
Overview of candidate genes for interaction partners of *DmManf* based on RNAi screen. UAS-*x*-RNAi lines were crossed to MS1096-GAL4 and 69B-GAL4 driver lines in wild type, heterozygous *DmManf* ^*Δ96*^ mutant and *DmManf* overexpression background. Observed phenotypes of knockdown flies in heterozygous *DmManf* ^*Δ96*^ mutant background (het vs. wt) and *DmManf* overexpression background (OE vs. wt) were compared to phenotype of knockdown flies in wild type background. *Yellow *(stronger phenotype) and *green* (rescued phenotype) represent affected phenotypes. *Light gray* (no phenotype), *gray* (phenotype not affected) and *dark gray* (lethal phenotype) represent cases where heterozygous *DmManf* ^*Δ96*^ mutant background or overexpression of *DmManf* did not affect the phenotype caused by knockdown of target gene. For a comparison, results from microarray analysis (MAA) [14] are presented; *red* and *blue* indicate up- and downregulation of target genes, respectively. Mutant larvae stands for zygotic *DmManf* ^*Δ96*^ mutant larvae, OE larvae for 69B-GAL4 > UAS-*DmManf* ^L3^ larvae, and mutant embryos for maternal and zygotic *DmManf* ^*Δ96*^ mutant embryos. Cellular compartment: Ch, chromosome; Cy, cytoplasm; Ec, extracellular; Me, membrane; Mi, mitochondrion; Nu, nucleus; nd, no data available; Sg, signalosome; Sp, spliceosome. Het, heterozygous *DmManf* mutant; MAA, microarray analysis; OE, overexpression

**Fig. 5:**
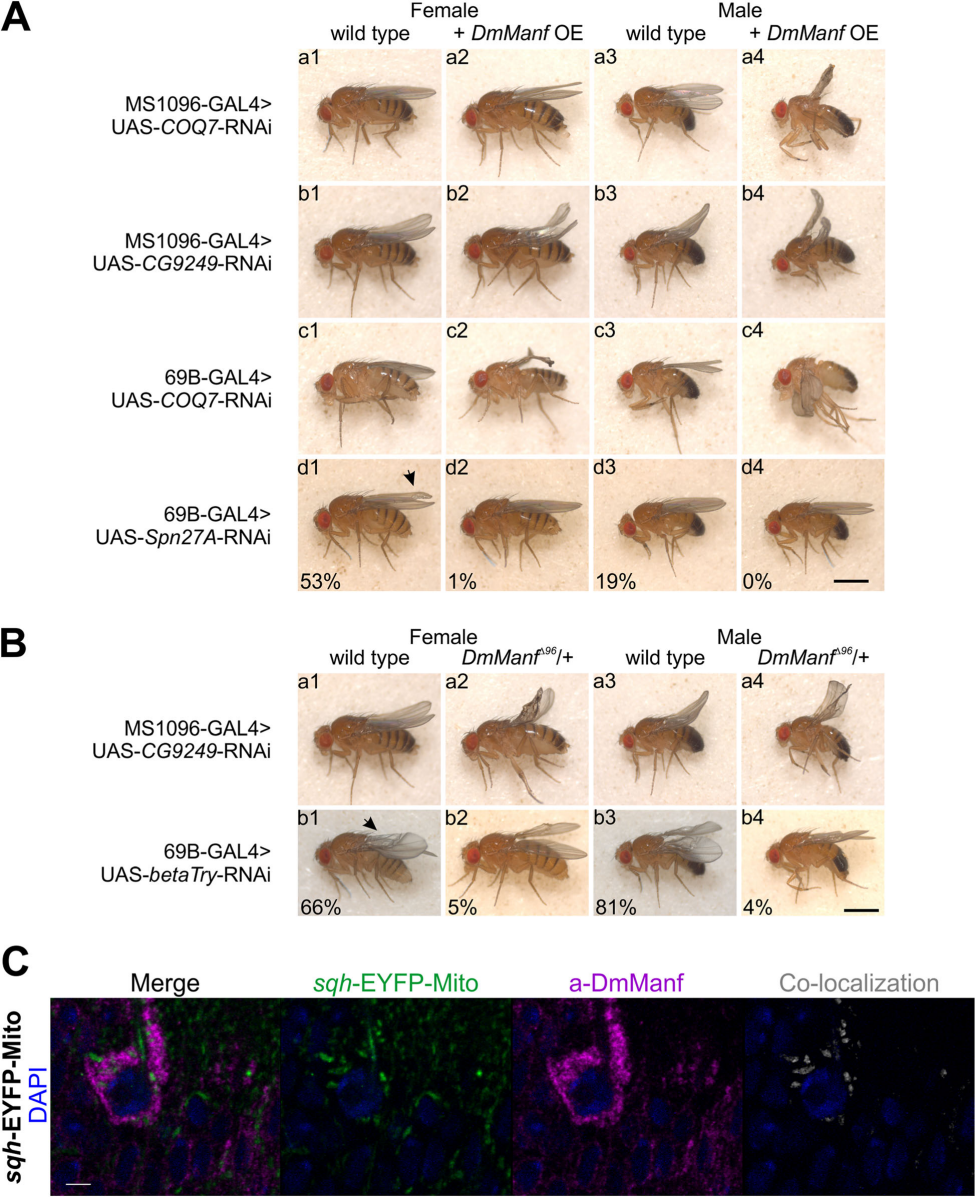
Knockdown of candidate genes is affected by genetically altered DmManf level. Overexpression of *DmManf* (**a**) and heterozygous *DmManf* ^*Δ96*^ mutant background (**b**) affect phenotypes of candidate gene knockdown with wing driver MS1096-GAL4 (a and b in **a**, a in **b**) and semi-ubiquitous driver 69B-GAL4 (c and d in **a**, b in **b**). The knockdown of *COQ7* and *CG9249* are discussed in the main text. Knockdown of *Spn27A* (Serpin-27A, an inhibitor of serine-type endopeptidase activity) with 69B-GAL4 resulted in distal blister phenotype (*arrow*) with incomplete penetrance (d1 and d3 in **a**). When *DmManf* was simultaneously overexpressed, the prevalence of blistered phenotype was significantly decreased (d2 and d4 in **a** and data not shown). Knockdown of *betaTry* (betaTrypsin, a serine-type endopeptidase involved in proteolysis) with 69B-GAL4 caused a blistered wing phenotype (*arrow*) with incomplete penetrance (b1 and b3 in **b**). The heterozygous *DmManf* ^*Δ96*^ mutant background significantly suppressed the prevalence of this blistered wing phenotype (b2 and b4 in **b** and data not shown). The percentages in d1-d4 in A and b1-b4 in B represent the proportion of adult flies with indicated phenotype. Number of analysed adults in (**a**): d1, 75; d2, 127; d3, 63; d4, 142; and in (**b**): b1, 325; b2, 189; b3, 286; b4, 236. Scale bar 1 mm. OE, overexpression. **c** Anti-DmManf (*magenta*) partially co-localized with mitochondrial marker *sqh*-EYFP-Mito (*green*) in the thoracic CNS of 3rd instar larvae. Images consist of four laser confocal sections. Nuclei are shown in blue and gray represents the co-localization of anti-DmManf and *sqh*-EYFP-Mito. See Additional file 5 for 3D volume rendering. Scale bar 3 μm

The observed wing phenotype allowed us to perform a straightforward genetic screen to identify novel genetic interactions of *DmManf*. Overall scheme of our four-staged RNAi screen is presented in Additional file 2. We screened approximately 2800 RNAi lines (representing about 20% of fly genome) from VDRC and National Institute of Genetics (NIG, Japan) collections. The RNAi lines were selected randomly to perform unbiased screening for the interacting partners. The primary screen was conducted with MS1096-GAL4 driver (E. Vridolin and O. Shimmi, unpublished data) and RNAi lines with similar wing phenotype to *DmManf*-RNAi were selected for secondary screening. MS1096-GAL4 is expressed in the pouch area of the wing disc (B in Additional File 2, [37–39]), but we also detected a weak expression pattern in central nervous system (CNS) (C in Additional File 2). For more efficient screening, we focused on the wing phenotypes only. The secondary screen was performed for approximately 400 RNAi lines with two GAL4 drivers, MS1096-GAL4 and semi-ubiquitous 69B-GAL4, in both wild type and heterozygous *DmManf* ^*Δ96*^ mutant backgrounds to observe whether the phenotype caused by silencing of a particular gene would be altered by decreased *DmManf* expression level [3]. 69B-GAL4 lacks expression in the fat body, gastric caeca and muscles, and its expression patterns in the CNS and cuticle are limited [10, 14]. In addition to its semi-ubiquitous expression pattern, we chose 69B-GAL4 driver because *DmManf* ^*Δ96*^ mutant lethality is fully rescued by ectopic expression of UAS-*DmManf* with 69B-GAL4 [3]. About 80 RNAi lines showed altered phenotype between wild type and heterozygous *DmManfΔ96* ^*Δ96*^ mutant background and were selected for tertiary screening. In tertiary screening, we used overexpression of *DmManf* together with knockdown of candidate genes with 69B-GAL4 and MS1096-GAL4 drivers to study whether the increased level of DmManf would affect the phenotypes caused by silencing of the candidate genes. The overexpression of *DmManf* with 69B-GAL4 or MS1096-GAL4 alone showed no detectable phenotype in adults (D in Additional file 2). Based on secondary and tertiary screening, we selected 21 genes as candidate interacting partners of *DmManf* (Fig. 4). Examples of candidate gene knockdown experiments are presented in Fig. 5a–b and Additional file 3.

The putative interactions between *DmManf* and the 21 candidate genes were further analysed by ubiquitous knockdown of the candidate genes (stage 4 in Additional file 2, B-C in Additional file 3, Additional file 4). We used *tub*-GAL4 driver and compared pupal and adult viability between wild type background and with *DmManf* overexpression to see whether overexpression of *DmManf* would affect the phenotypes caused by ubiquitous knockdown of the candidate genes. For example, simultaneous overexpression of *DmManf* significantly decreased the pupal viability for ubiquitous knockdown of *CSN3* (COP9 complex homolog subunit 3) encoding a protein involved in regulation of the ubiquitin conjugation pathway (B-C in Additional file 3). Overexpression of *DmManf* alone with *tub*-GAL4 in wild type background showed no obvious phenotype or affected fly viability [13].

### Gene ontology terms of potential *DmManf* interacting partners

We analysed the Gene Ontology (GO) terms of the 21 candidate genes (Table 2). Four of the candidate genes were unannotated and excluded from the analysis. Most enriched GO terms were related to ubiquinone related processes (2 genes), mitochondrial cellular compartment (6 genes) and cellular metabolic process (13 genes). Alterations in genes annotated with GO terms related to metabolism were also observed in our previous microarray analysis [14]. The enrichment of mitochondrial genes was notable: 29% of our candidate genes were annotated with mitochondria as cellular compartment while according to FlyBase [40], only 5% of all *Drosophila* genes have such annotation (http://gostat.wehi.edu.au/). Furthermore, three of our candidate genes (*Ts*, *l(2)37Bb* and *CG9249*) with unknown cellular compartment have human homologues which are annotated to mitochondria.

**Table 2:** Enrichment of *Drosophila* GO terms in the partial RNAi screen

GO ID	GO term	Genes	Count	Total	*P*-Value
GO:0006743	ubiquinone metabolic process	*COQ7, COQ3*/*CG9249*	2	2	0.013
GO:0006733	oxidoreduction coenzyme metabolic process	*COQ7, COQ3*/*CG9249*	2	2	0.013
GO:0042375	quinone cofactor metabolic process	*COQ7, COQ3*/*CG9249*	2	2	0.013
GO:0031966	mitochondrial membrane	*COQ7, ND75, Tom20, CG6455*	4	34	0.033
GO:0005740	mitochondrial envelope	*COQ7, ND75, Tom20, CG6455*	4	37	0.035
GO:0005739	mitochondrion	*COQ7, Hsp68, Aats-phe, ND75, Tom20, CG6455*	6	106	0.035
GO:0031975	envelope	*COQ7, ND75, Tom20, CG6455*	4	47	0.058
GO:0031967	organelle envelope	*COQ7, ND75, Tom20, CG6455*	4	47	0.058
GO:0044237	cellular metabolic process	*betaTry, COQ7, dome, Hsp68, Ku70*/*Irbp, ND75, Aats-phe, pABp, Spn27A, Taf5, Ts, Cdk12*/*CG7597, COQ3*/*CG9249*	13	569	0.064
GO:0031090	organelle membrane	*COQ7, ND75, Tom20, CG6455*	4	54	0.078

Analysis of Gene Ontology (GO) terms was performed on 21 candidate genes by GOstat (http://gostat.wehi.edu.au/). GO IDs with *P*-value <0.1, corresponding GO terms and list of genes are presented. Count, number of our candidate genes mapping to a GO term; Total, number of genes in our primary screen annotated with each GO term

### DmManf partially localizes to mitochondria and genetically interacts with the ubiquinone synthesis pathway

To examine further the interaction between *DmManf* and genes encoding mitochondrial proteins, we studied the subcellular localization of DmManf. According to our previous analyses, DmManf is localized to several cell compartments [14]. To detect mitochondria, we used *sqh*-EYFP-Mito transgenic fly line [41]. When the CNS of 3rd instar *sqh*-EYFP-Mito larvae were immunohistochemically stained with DmManf antibody, a partial co-localization with mitochondrial marker was detected (Fig. 5c and Additional file 5).


Additional file 5: Video of 3D volume rendering of co-localization of a-DmManf and *sqh*-EYFP-Mito. A mov file. DmManf expression (magenta) adjoined the *sqh*-EYFP-Mito marker (green). (MOV 6408 kb)


**Fig. 6:**
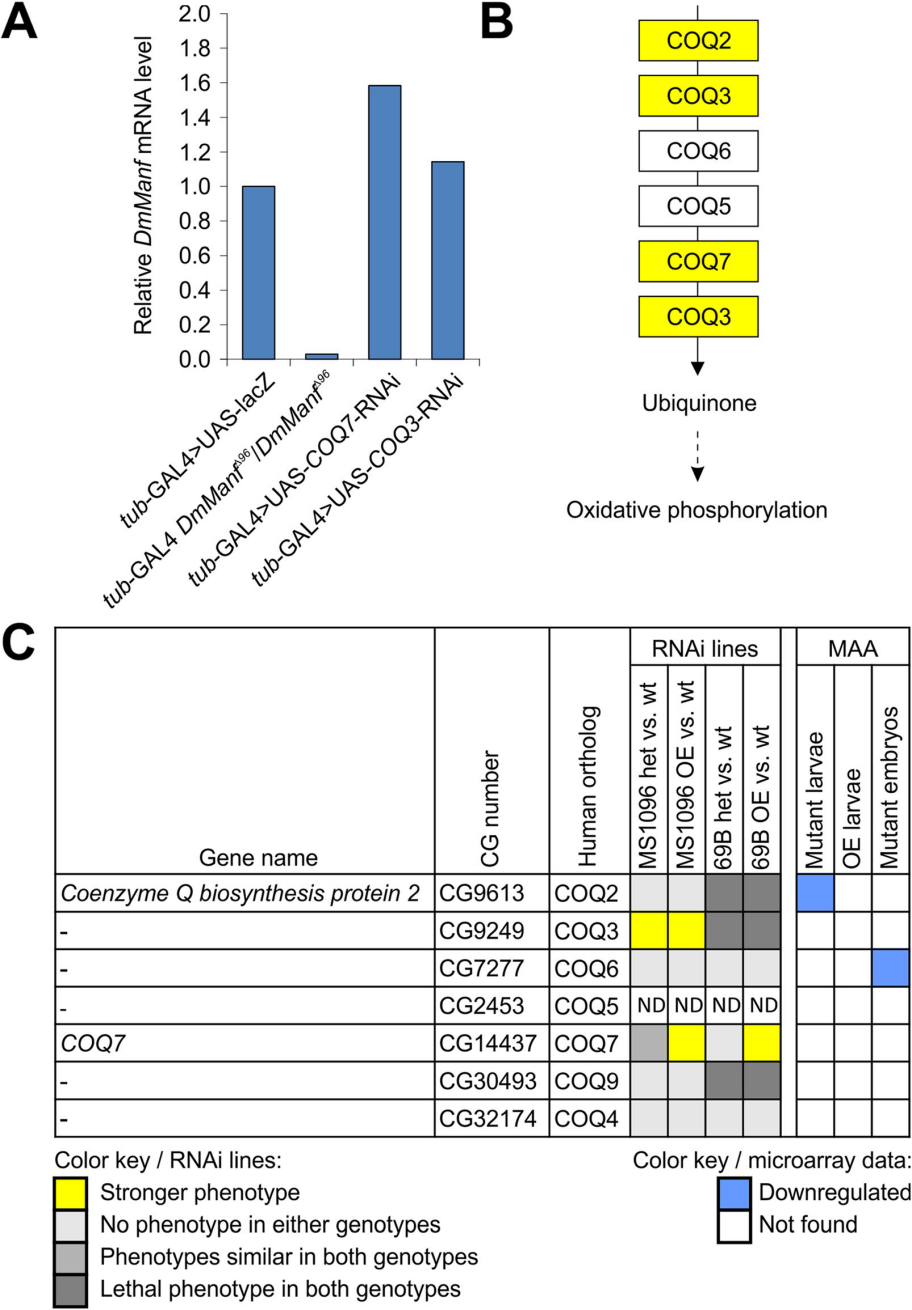
*DmManf* interacts with genes involved in the ubiquinone synthesis pathway. **a** Quantitative RT-PCR analysis showed increased *DmManf* mRNA level when *COQ7* was ubiquitously knocked down with *tub*-GAL4. UAS-*lacZ* was used as a wild type control. **b** A schematic presentation of ubiquinone synthesis pathway according to Kyoto Encyclopedia of Genes and Genomes (http://www.genome.jp/kegg/, pathway ID 00130). Genes marked with yellow were found to genetically interact with *DmManf*. **c** UAS-RNAi lines of genes involved in ubiquinone synthesis pathway were crossed to MS1096-GAL4 and 69B-GAL4 drivers in wild type, heterozygous *DmManf* ^*Δ96*^ mutant or *DmManf* overexpression background. See Fig. 4 legend for interpretation and Additional file 2 for schematic presentation of the crosses. Het, heterozygous *DmManf* ^*Δ96*^ mutant; MAA, microarray analysis; ND, not determined; OE, overexpression

Two of our candidate genes were involved in the ubiquinone biosynthesis pathway, *COQ7* (coenzyme Q7 homologue) and *CG9249* (homologue of coenzyme Q3, COQ3) (Fig. 4 and Fig. 6). Ubiquinone is an electron carrier involved in respiratory electron transport chain localized to inner mitochondrial membrane (reviewed in [42, 43]). The knockdown of *COQ7* caused more severe phenotype with overexpression of *DmManf* than in wild type background when MS1096-GAL4, 69B-GAL4 or ubiquitous *tub*-GAL4 drivers were used. More specifically, the silencing of *COQ7* with MS1096-GAL4 led to very mildly bent-up wings in wild type background (a1 and a3 in Fig. 5a). With simultaneous overexpression of *DmManf* this phenotype was stronger leading to small, wrinkled wings in adult males (a4 in Fig. 5a). The knockdown of *COQ7* with 69B-GAL4 led to mildly uneven surface of the wing in wild type background but to a wrinkled wing phenotype when *DmManf* was overexpressed (c1-c4 in Fig. 5a). When *COQ7* was ubiquitously knocked down with *tub*-GAL4 in wild type background, lethality occurred during pupal stage and no adults emerged (B-C in Additional file 3). However, simultaneous overexpression of *DmManf* led to complete lethality at larval stage (B in Additional file 3). Ubiquitous knockdown of *COQ7* with *tub*-GAL4 increased *DmManf* mRNA level determined by qPCR analysis (Fig. 6a).

Knockdown of *COQ3/CG9249* with 69B-GAL4 and *tub*-GAL4 led to lethality at pupal and larval stages, respectively, and was not affected by either heterozygous *DmManf* ^*Δ96*^ mutant background or overexpression of *DmManf* (Fig. 4 and Additional file 4). When *COQ3/CG9249* was knocked down with MS1096-GAL4 in wild type background, a bent-up phenotype was observed in the adult wing (b1 and b3 in Fig. 5a, a1 and a3 in Fig. 5b). This phenotype was stronger leading to wrinkled wings when either simultaneous overexpression of *DmManf* (b1-b4 in Fig. 5a) or heterozygous *DmManf* ^*Δ96*^ mutant background (a1-a4 in Fig. 5b) was used. Ubiquitous knockdown of *COQ3/CG9249* with *tub*-GAL4 showed a very slight increase in *DmManf* mRNA levels detected by qPCR analysis (Fig. 6a).

In *Drosophila*, only six genes are annotated to be involved in processes related to the ubiquinone synthesis pathway (GO:0006744 term in FlyBase, http://flybase.org; Fig. 6b). We decided to study interaction between *DmManf* and the rest of the ubiquinone synthesis pathway genes, *COQ2/CG9613*, *COQ6/CG7277*, *COQ9/CG30493*, and *COQ4/CG32174* (Fig. 6b–c). Wing-specific knockdown of any of these genes with MS1096-GAL4 showed no phenotype (Fig. 6c). Knockdown with semi-ubiquitous 69B-GAL4 driver showed either no phenotype (in the case of *COQ6* and *COQ4*) or led to lethality at pupal stage (*COQ9* and *COQ2*) and observed phenotype was not altered by either heterozygous *DmManf* ^*Δ96*^ mutant background or simultaneous overexpression of *DmManf* (Fig. 6c). Simultaneous overexpression of *DmManf* did not affect the observed phenotype by ubiquitous knockdown of *COQ6*, *COQ9* and *COQ4* with *tub*-GAL4. However, we observed significantly decreased pupal viability when *COQ2* was ubiquitously knocked down by *tub*-GAL4 in DmManf-overexpressing background compared to the wild type background (B in Additional file 3; Additional file 4).

### *DmManf* genetically interacts with *Irbp*, the *Drosophila* homologue of *Ku70*

**Fig. 7:**
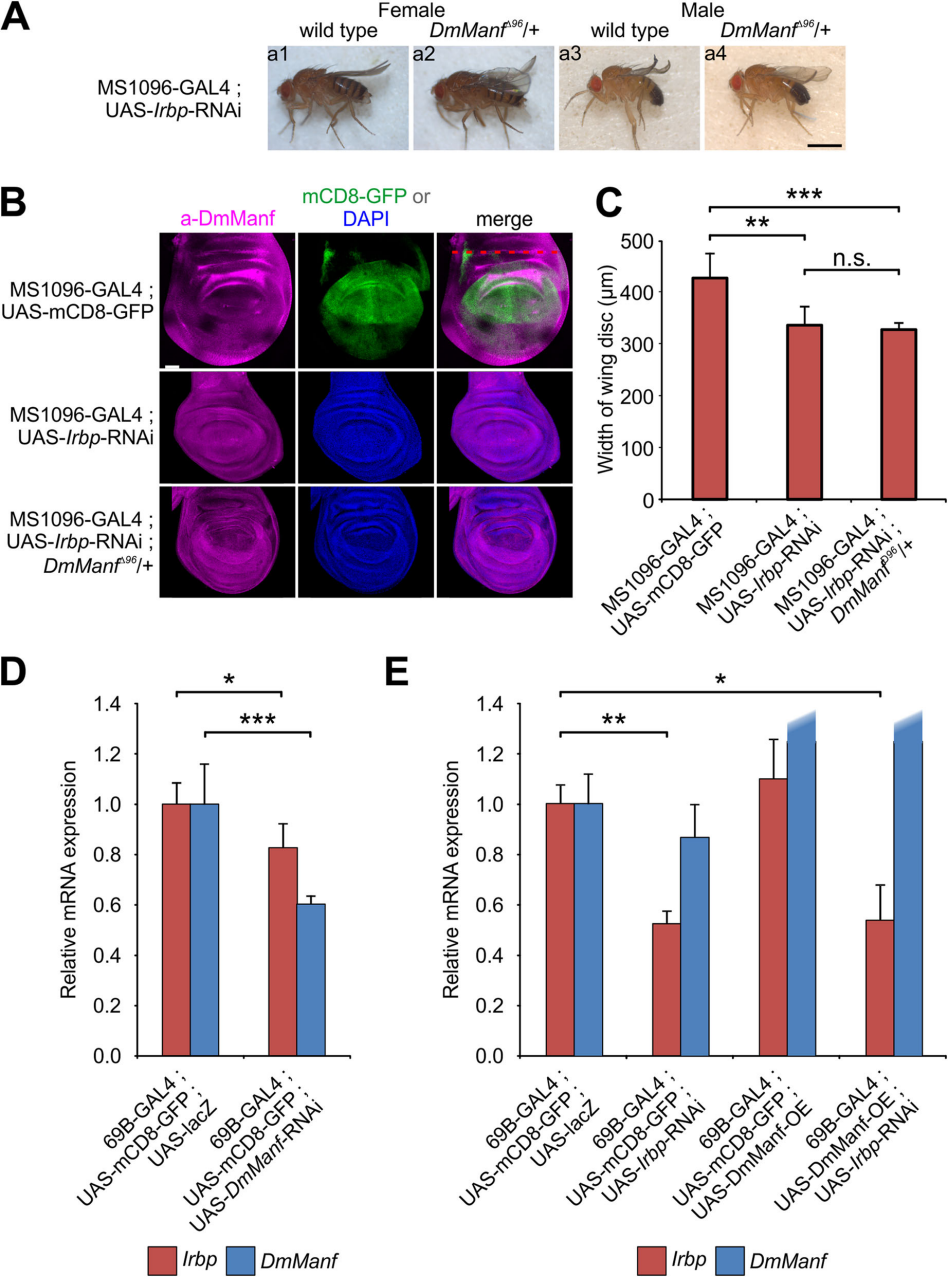
The *Drosophila* homologue of Ku70, *Irbp*, is a genetic interacting partner of *DmManf*. **a** Heterozygous *DmManf* ^*Δ96*^ mutant background affected phenotype of *Irbp* knockdown with wing driver MS1096-GAL4 in adult male flies (a3-a4). Scale bar 1 mm. **b**-**c** The wing discs of *Irbp* knockdown with MS1096-GAL4 showed significantly decreased width (red dashed line) in comparison to control genotype (MS1096-GAL4; UAS-mCD8-GFP). *n* = 8 (control), *n* = 5 (*Irbp*-RNAi in wild type background), *n* = 7 (*Irbp*-RNAi in heterozygous *DmManf* ^*Δ96*^ mutant background). **, *P* < 0.01, ***, *P* < 0.001, Student’s t-test. n.s., not significant. Scale bar 50 μm. **d**-**e** Knockdown embryos of *DmManf* with 69B-GAL4 showed decreased *DmManf* and *Irbp* mRNA levels (**d**) and *Irbp* mRNA expression was decreased in 3rd instar larvae of *Irbp* knockdown with 69B-GAL4 analysed by qPCR (**e**). Overexpression of DmManf did not affect *Irbp* mRNA expression. *DmManf* mRNA expression was not altered in *Irbp* knockdown with 69B-GAL4. Overexpression of DmManf with 69B-GAL4 increased *DmManf* mRNA level (relative mRNA expression >30; not shown). *, *P* < 0.05, **, *P* < 0.01, ***, *P* < 0.001, Student’s t-test

Three dimensional structure of C-terminal domain of human MANF shows the highest similarity with the SAP domain of Ku70, an inhibitor of Bax-induced cell death [22, 23]. Microinjected MANF encoding cDNA or protein has also been shown to protect cultured mouse superior cervical ganglion neurons from drug induced apoptosis [10, 22]. In our genetic screen, we identified *Irbp* (Inverted repeat-binding protein), a homologue of mammalian Ku70, as a potential interacting partner of *DmManf* (Fig. 4). In adult male flies, knockdown of *Irbp* with MS1096-GAL4 driver in wild type background showed a wrinkled wing phenotype (a3 in Fig. 7a). When UAS-*Irbp*-RNAi was driven with MS1096-GAL4 in heterozygous *DmManf* ^*Δ96*^ mutant background, male flies showed a bent-up wing phenotype, clearly milder than in wild type background (a4 in Fig. 7a). In female flies, no alteration between wild type and heterozygous *DmManf* ^*Δ96*^ mutant background was detected (a1-a2 in Fig. 7a).

We noted that *Irbp* knockdown with MS1096-GAL4 resulted in smaller wing discs of 3rd instar wandering larvae in comparison to wild type (Fig. 7b). We quantified the width of the wing disc above the wing pouch area (indicated by red dashed line in Fig. 7b) and found that in the *Irbp* knockdown flies the width of the wing disc was significantly decreased in comparison to wild type (Fig. 7c). We also studied whether the heterozygous *DmManf* mutant background would affect the width of the wing disc in *Irbp* knockdown larvae with MS1096-GAL4 and thus explain the altered wing phenotype of *Irbp* knockdown flies in this background. However, there was no difference in the width of the wing disc when *Irbp* was knocked down in wild type and heterozygous *DmManf* mutant backgrounds (Fig. 7c).

We observed lethality at pupal stage when *Irbp* was ubiquitously knocked down with *tub*-GAL4 (B in Additional file 3). Simultaneous overexpression of *DmManf* partially rescued this lethality to adulthood (B-C in Additional file 3) suggesting that DmManf and Irbp may act in the same pathway and have, to certain extent, redundant function.

To examine whether altered expression level of *Irbp* and *DmManf* affect each other, we collected embryos of *DmManf* knockdown as well as wandering 3rd instar larvae of *Irbp* knockdown and DmManf overexpression with semi-ubiquitous 69B-GAL driver and quantified *Irbp* and *DmManf* mRNA levels by qPCR analysis. In *DmManf* knockdown embryos, *DmManf* mRNA expression level was significantly decreased in comparison to the control (Fig. 7d). Interestingly, *Irbp* mRNA expression was also significantly decreased in *DmManf* knockdown embryos (Fig. 7d) indicating that loss of *DmManf* downregulated *Irbp* expression.

In *Irbp* knockdown larvae *Irbp* mRNA level was significantly decreased while *DmManf* mRNA expression was not altered (Fig. 7e). In DmManf-overexpressing larvae, the level of *Irbp* mRNA did not differ from the control genotype (Fig. 7e). When *Irbp* knockdown was done in wild type and DmManf-overexpressing backgrounds, the decreased *Irbp* mRNA level was not affected (Fig. 7e) indicating that the overexpression of DmManf does not neither induce nor repress *Irbp* mRNA expression. We also studied the effect of *Irbp* knockdown on DmManf protein expression by investigating wing discs of *Irbp* knockdown 3rd instar larvae. In line with our qPCR analysis, DmManf immunoreactivity was not altered in *Irbp* knockdown with MS1096-GAL4 in comparison to wild type (Fig. 7b).

Based on the structural homology between the Bax-inhibiting SAP domain of Ku70 and C-terminal domain of MANF [22, 23], we hypothesized that the total loss of DmManf in vivo could lead to decreased inhibition of Bax followed by increased cell death and subsequent *DmManf* ^*Δ96*^ mutant lethality. Thus, we tested whether the loss of *Debcl* (death executioner Bcl-2 homologue), a homologue to mammalian proapoptotic Bax subfamily, could rescue the *DmManf* ^*Δ96*^ mutant lethality in vivo. We used two *debcl* mutant alleles (loss-of-function allele *debcl* ^*E26*^ and putative dominant negative allele *debcl* ^*W105*^) to abolish endogenous Debcl [44] and UAS-*debcl*-RNAi line together with ubiquitous *da*-GAL4 driver to knock down *Debcl*. However, neither the loss nor silencing of Debcl could rescue the *DmManf* ^*Δ96*^ mutant lethality (Additional file 6) suggesting that during development DmManf action through other molecular systems than Debcl is more crucial for viability.

## Discussion

MANF and CDNF form an evolutionarily conserved family of neurotrophic factors [1–3]. Since their discovery, increasing data suggests that these proteins possess other characteristics beyond their neurotrophic properties. The loss of *Drosophila* homologue, *DmManf*, results in lethality at early developmental stages with neuronal and cuticular defects [3]. Thus, we wanted to explore the tissue-specific effects of knockdown of *DmManf* using UAS-RNAi approach. In a previous study, neither neuronal nor glial knockdown of *DmManf* showed any obvious phenotypic effects without the overexpression of Dicer-2, a component of RNAi machinery [45]. Here, we demonstrated that knockdown of *DmManf* by UAS-*DmManf*-RNAi construct is effective and specific. The ubiquitous knockdown resembled the lethal phenotype of homozygous *DmManf* ^*Δ96*^ mutants. We detected a phenotype in the wing when knocking down *DmManf* with a wing-specific driver MS1096-GAL4. The wing phenotype was stronger in heterozygous *DmManf* ^*Δ96*^ mutant background as compared to the wild type background further demonstrating the specificity of UAS-*DmManf*-RNAi construct. Although the phenotype of small or even absent wings observed in the *DmManf* knockdown adult males would suggest a decrease in cell number, the number of mitotic cells was significantly increased in the wing disc of *DmManf* knockdown larvae. Our results are in accordance with the previous study showing that the silencing of human MANF in HeLa cells stimulated cell proliferation [17]. The increased appearance of the mitotic markers used in this study could also be due to prolonged M phase of cell cycle. This would be in line with our finding that the density of cells was not altered between wild type and *DmManf* knockdown larval wing discs (data not shown). Prolonged cell cycle would result in decreased growth rate and could explain the wing phenotype observed in *DmManf* knockdown flies. Furthermore, we failed to detect any differences with apoptotic markers (data not shown) indicating that the wing phenotype did not result from increased cell death at the 3rd instar larval stage. However, it is possible that apoptosis could take place later at the pupal stage.

The knowledge on the molecular mechanisms and signaling pathways of MANF/CDNF family proteins is still limited. Previous studies show strong evidence supporting MANF function in ER stress and unfolded protein response [8, 9, 15, 17, 20]. In our recent study, we showed that the expression of *DmManf* mRNA is upregulated in response to ER stress-inducing drugs and that *DmManf* genetically interacts with genes known to function in ER stress and unfolded protein response [13]. In the current work we utilized the wing phenotype detected in *DmManf* knockdown flies and performed a partial unbiased screen of two genome-wide UAS-RNAi libraries using the *Drosophila* in vivo model. The primary screen was done with wing-specific driver (MS1096-GAL4) and the selection of candidate genes was based solely on phenotypic similarity to *DmManf*-knockdown flies. We were unable to find genes with ER- and ER stress-related functions as potential interaction partners for DmManf because of this criterion: knockdown of ER stress related genes with MS1096-GAL4 driver resulted in distinct phenotypes in comparison to the knockdown of *DmManf* [13]. In the secondary and tertiary screens we knocked down the candidate genes in (1) heterozygous *DmManf* ^*Δ96*^ mutant background to decrease the level of endogenous DmManf protein and (2) with simultaneous overexpression of *DmManf* to increase it [3]. Thus, we aimed to discover whether the phenotype caused by silencing of a particular gene would be affected by manipulating *DmManf* expression level.

Although our screen was only partial, it strongly suggested that *DmManf* interacts with genes encoding mitochondrial proteins. Mitochondria play crucial roles e.g. in oxidative phosphorylation and Ca^2+^ signaling, and their dysfunctional biogenesis and metabolism are involved in a variety of human diseases (reviewed in [46–48]). We found that DmManf partially co-localized with the mitochondrial marker *sqh*-EYFP-Mito. Deeper analysis revealed that strongest DmManf expression was detected adjoining the mitochondrial marker. In *sqh*-EYFP-Mito marker, the fluorophore is directed to mitochondria by the signal peptide of human Cox8A (cytochrome C oxidase subunit 8A) and localized to the mitochondrial matrix [41]. Since DmManf is also localized to ER [10, 14], the observed co-localization could be on the membranes connecting ER and mitochondria. DmManf could take part in the ER-mitochondrial crosstalk and disturbances in DmManf protein levels could affect protein transport to mitochondria. A specific complex, TOM (translocase of outer membrane), is needed for proper targeting of mitochondrial proteins encoded by nuclear DNA (reviewed in [49]). We identified genetic interaction between *DmManf* and *Tom20* (Translocase of the outer membrane 20, homologue to human TOMM20 and TOMM20-L), a receptor subunit of TOM. Interestingly, another receptor protein of TOM, Maggie/TOMM22 has been shown to mediate localization of pro-apoptotic Debcl (homologue of mammalian Bax) to mitochondria in *Drosophila* [50]. Bax, together with other members of the Bcl-2 protein family, regulates permeabilization of mitochondrial outer membrane during apoptosis (reviewed in [51]). Previous studies have revealed a role for MANF in Bax-induced cell death in vitro [10, 22]. In addition, our screen data suggests a genetic interaction between MANF and Ku70/Irbp, an inhibitor of Bax/Debcl. In future, it would be interesting to evaluate whether DmManf co-localizes with TOM proteins and whether Irbp or Debcl have any role in this interaction.

Two genes from the ubiquinone synthesis pathway, *COQ7* and *CG9249*/*COQ3*, were included in our primary screen and identified as candidate interacting partners of *DmManf*. We also found evidence for interaction between *DmManf* and a third component of ubiquinone synthesis pathway, *CG9613/COQ2*. The knockdown of *COQ7* or *CG9249*/*COQ3* resulted in elevated *DmManf* mRNA levels. The best known function of ubiquinone, also known as coenzyme Q (Q), is its participation in electron transport chain (ETC) by transferring electrons from complexes I and II to complex III (reviewed in [42, 43, 52]). COQ7 hydroxylates demethoxyubiquinone (DMQ) into hydroxyquinone [53] from which ubiquinone is formed by COQ3 [54]. Loss of COQ7 (also known as Mclk-1 in mice, clk-1 in nematodes) leads to Q deficiency and impaired ATP synthesis [55–58]. Q deficiency in humans (OMIM 607426) is associated with variety of clinical manifestations, mostly neuronal and muscular defects (reviewed e.g. in [59]). Importantly, increasing evidence suggests that mitochondrial dysfunction is one of the main causes of PD (reviewed e.g. in [48]) and studies on neuroprotection by Q treatment in PD models have been promising (reviewed in [60]). Q deficiency has been linked to destabilization of mitochondrial complex I [61]. Complex I is also associated with PD as mutations in its subunits are found to be involved in a familial form of PD (OMIM 556500; reviewed in [48]). Furthermore, toxins used to induce PD-like symptoms in animal models include MPTP, rotenone and paraquat which all interfere with complex I functionality [62, 63]. In addition to genes involved in Q synthesis, we found homologues for two genes linked to human mitochondrial complex I deficiency (OMIM 252010), *ND75* (NDUFS1) and *l(2)37Bb* (FOXRED1) to genetically interact with *DmManf*. Considering all our data indicating a mitochondrial function, DmManf could affect the oxidative phosphorylation, directly or indirectly. In future studies, the connection between DmManf protein and its function in mitochondria should be thoroughly examined.

Alternatively, DmManf could play a role in maintaining cellular Ca^2+^ homeostasis, an important function of both mitochondria and ER, based on Ca^2+^-dependent binding of mammalian MANF and GRP78 [8]. In our previous study, cultured Schneider 2 -cells showed a strong induction of *DmManf* mRNA expression in response to thapsigargin, an inhibitor of ER membrane-resident Ca^2+^ ATPase, which depletes Ca^2+^ from ER [13]. Additionally, one of our candidate genes, *CG6455*, is homologous to human IMMT (inner membrane protein, mitochondrial) predicted with a function in mitochondrial Ca^2+^ homeostasis.

For several of our candidate genes, e.g. *CG9249*/*COQ3*, knockdown with MS1096-GAL4 in both *DmManf* ^*Δ96*^ heterozygous mutant (with decreased DmManf protein level) and overexpression (with increased DmManf protein level) background resulted in more severe phenotype in comparison to the phenotype observed in wild type background. This suggests that knockdown of certain genes together with the imbalance of DmManf protein level affects overall cellular homeostasis rather than disturbs a putative stoichiometric relationship between DmManf and candidate gene encoded protein levels. Furthermore, while the genetic interaction discovered may represent a physical or biochemical interaction, it might also indicate a secondary effect resulting from involvement of *DmManf* and candidate gene in the same signaling pathway or biological process.

## Conclusions

This study revealed that DmManf is involved in *Drosophila* wing development and expanded our knowledge on the role of MANF in the maintenance of cellular homeostasis. Importantly, we discovered novel genetic interacting partners of DmManf and our study suggests that MANF has a role in mitochondrial function. These data help us understand the molecular mechanism of the evolutionarily conserved MANF/CDNF protein family in future studies.

## Methods

### Fly strains and antibodies

Fly stocks and crosses were maintained at 25 °C. The following fly lines were used in the study: *w* ^−^, UAS-*DmManf* ^133^ (line L3), UAS-*DmManf* ^135^ (line L5) and *DmManf* ^*Δ96*^/TM6 Tb Sb EYFP [3], UAS-*HsMANF* ^L2^ and UAS-*HsCDNF* ^L1^ [10], UAS-*lacZ* [31]. The following lines were obtained from Bloomington *Drosophila* Stock Center: 69B-GAL4 (#1774, [31]), A9-GAL4 (#8761, [38]), *da*-GAL4 (#5460, [64]), *en*2.4-GAL4^e16E^ (#30564, A. Brand & K. Yoffe, unpublished), MS1096-GAL4 (#8860, [37]), *salm*-GAL4 (#5818, [65]), *Ser*-GAL4 (#6791, [66]), *tub*-GAL4/TM6 Tb Sb EYFP (#5138) and UAS-mCD8-GFP (#5130) [67], *debcl* ^*E26*^ (#27342) and *debcl* ^*W105*^/CyO (#27341) [44], *act*-His2Av-mRFP (#23651, [68]), UAS-GFP.nls (#4775, [31]) and *sqh*-EYFP-Mito (#7194, [41]). UAS-S/G2/M-Green was obtained from Kyoto Stock Center (#109676, [36]). Combination of *act*-His2Av-mRFP and UAS-S/G2/M-Green in 2nd chromosome was a kind gift from Jinghua Gui. T(2;3)SM6a-TM6B Tb translocation balancer was used in viability studies (referred as SM6-TM6). UAS-RNAi lines were obtained from Vienna *Drosophila* RNAi Center and National Institute of Genetics (Additional file 7). Adult flies were imaged with ProgRes SpeedXT camera (Jenoptik). The following antibodies were used: rabbit anti-DmManf [3], rabbit anti-phospho-Histone H3 (Ser10) (06–570, Upstate), anti-α-tubulin (DM1A, Sigma).

### Immunohistochemistry, confocal microscopy and image analysis

Third instar larval wing discs and CNS were dissected in PBS and fixed with 4% paraformaldehyde in PBS or PEM (100 mM PIPES pH 7.0, 2 mM EGTA, 1 mM MgSO_4_) for 30 min. Fixed tissues were washed with PBT (0.1% Triton X-100 in PBS) and blocked with blocking solution (1% BSA in PBT) for 1 h. Tissues were incubated with primary antibody overnight at 4 °C and with secondary antibody for 1 h in room temperature, and mounted in VECTASHIELD® Mounting Medium (Vector Laboratories). Samples were imaged with TCS SP5 laser scanning microscope (Leica Microsystems) equipped with HCX PL APO 20×/0.7 mm Imm Corr glycerol immersion objective or HC PL APO 10×/0.4 air objective. For co-localization study, Zeiss LSM5 DUO confocal microscope equipped with PL APO 100×/1.4 oil objective was used. ImageJ 1.43u [69], Imaris 7.6.0 and Imaris 8.4.1 (Bitplane Inc.) were used for image analysis. For quantification of pHis3 positive cells, automatic “Spots” algorithm in Imaris 7.6.0 was used. For quantification of S/G2/M-Green and *act*-His2Av-RFP positive cells, a 37.9 μm × 37.9 μm area of the dorsal wing pouch was analyzed with the Spots algorithm in Imaris 8.4.1.

### Western blot analysis

Lysis buffer (20 mM Tris-HCl pH 7.4, 150 mM NaCl, 1% Triton X-100, 1 mM EDTA) supplemented with Complete proteinase inhibitor tablets (Roche) was used in homogenization of larvae. Western blotting was done according to manufacturer’s instructions and visualized by the Odyssey infrared imager (Li-Cor).

### Adult wing preparations

Adult flies were dipped into 70% ethanol and fixed 10 min in clove oil (Sigma). Wings were dissected and mounted in 70% Canada Balsam/30% xylene. Nikon SMZ1500 was used for imaging.

### Quantitative RT-PCR

Larvae were grown at 25 °C on apple juice plates and collected 50–54 h after egg laying. For 3rd instar larval samples wandering larvae were collected from the vials. Embryos were collected from apple juice plates 16–22 h after egg laying. NucleoSpin® RNA II (Macherey-Nagel) was used in extraction and purification of total RNA. DNase treatment was done on-column according to the manufacturer’s instructions. First strand cDNA was synthesized from total RNA (1 μg) using RevertAid Premium Reverse Transcriptase (Thermo Scientific) and Oligo(dT_18_) primer at 53 °C according to manufacturer’s instructions. Expression of *DmManf* mRNA was quantified by LightCycler® 480 Real-Time PCR System with Lightcycler 480 SYBR Green I master mix (Roche) with primers DmManf forward 5′-AATCTGCGACCTTCGCTATG-3’and DmManf reverse 5′-TCGTTGAGGATTTTCTTCAGG-3′ [14]. *Irbp* was amplified with primers Irbp forward 5′-AGTTCATCACGTTGTCAAGAGC-3′ and Irbp reverse 5′-TACGATCGGACAGGATTTCG-3′ [70]. *RpL32* was amplified as a reference gene with primers RpL32 forward 5′-CGGATCGATATGCTAAGCTGT-3′ and RpL32 reverse 5′-GCGCTTGTTCGATCCGTA-3′ [14]. PCR efficiency (E) of each primer pair was determined from a relative standard curve. For *DmManf*, E = 1.98; for *Irbp*, E = 2.00; for *RpL32*, E = 1.97. Equation E^-Cp^ in which Cp indicates a crossing point was used to calculate relative concentration of *DmManf*, *Irbp* and *RpL32* mRNA in each sample. To present the results, the concentration of *DmManf* and *Irbp* was normalized to the level of *RpL32*. Each sample was analysed as a duplicate.

### Statistical analysis

Means were compared by Student’s t-test, null hypothesis was rejected at *P* < 0.05. Statistical analyses were performed by using Microsoft® Excel Analysis ToolPak (Microsoft® Office Professional Plus 2010). For pupal viability studies, normal (Tubby^+^; Tb^+^) and squat (Tb^−^) pupae were counted, the number of Tb^+^ pupae was divided by the number of all pupae and normalized to experimentally determined ratio from *tub*-GAL4/TM6 Tb Sb cross to wild type and to wild type balanced against SM6-TM6 translocation balancer (Additional file 4 and Additional file 8, wild type and *DmManf* overexpression data previously reported in [13]). For preliminary analyses two vials were counted and statistical analysis was done based on six vials with minimum of 40 pupae. For genotypes showing incomplete penetrance of the wing phenotypes, quantification of the penetrance was performed by counting adult flies with and without phenotype from 4 vials per genotype.

### Gene ontology analysis

Gene Ontology (GO) analysis was performed for genes considered as hits from our UAS-RNAi screen (21 genes) against the set of genes included in the primary screen (approximately 2800 randomly selected genes). GOstat (http://gostat.wehi.edu.au/) was used with default tool settings. A complete list of overrepresented (*p* < 0.1) GO terms is presented in Table 2.

## Additional files

Additional file 1: Two *DmManf*-RNAi constructs in three independent transformant lines are available in VDRC. A tiff file. A) Alignment of *DmManf*-RNAi constructs 4793 and 108792. Construct 4793 targets last three exons of the *DmManf* gene with no predicted off-targets (VDRC data sheet). Construct 108792 targets exon 4 and 3′ UTR and has one predicted off-target (*Sulfated*/*CG6725*; VDRC data sheet). Construct 4793 in transformant line 12835 was used in further studies. B) Different UAS-*DmManf*-RNAi transformant lines show similar phenotypes when driven with wing-specific MS1096-GAL4. For all lines, heterozygous *DmManf* ^*Δ96*^ mutant background lead to stronger wing phenotype and overexpression (OE) of *DmManf* rescued the wing phenotype. C) Alignment of UAS-*DmManf*-RNAi construct (ID 4793) with UAS-*HsMANF* and UAS-*HsCDNF* constructs. Strongest alignment is shown in green (*HsMANF*) and purple (*HsCDNF*). VDRC, Vienna *Drosophila* RNAi Center; Tf, transformant line; OE, overexpression. (TIFF 2536 kb)

Additional file 2: Screen for genetic interaction partners of *DmManf*. A tiff file. A) Scheme of the crosses used in the partial RNAi library screen. First, randomly selected UAS-*x*-RNAi lines were crossed to wing-specific driver line MS1096-GAL4 (1). Lines showing similar phenotype to UAS-*DmManf*-RNAi lines were selected to the next stage. Second, two GAL4 drivers, wing-specific MS1096-GAL4 and semi-ubiquitous 69B-GAL4, were used in both wild type and heterozygous *DmManf* ^*Δ96*^ mutant background (2) – UAS-*x*-RNAi lines showing distinct phenotypes in wild type and heterozygous *DmManf* ^*Δ96*^ mutant backgrounds were selected. Secondary stage was repeated for selected UAS-*x*-RNAi lines in order to ensure the observed interactions. At stage 3, 69B-GAL4 and MS1096-GAL4 drivers were used to express UAS-*x*-RNAi lines with or without UAS-*DmManf* overexpression construct (3). Based on stages 2 and 3, 21 UAS-*x*-RNAi lines were selected as candidates for final stage 4. (4) UAS-*x*-RNAi lines were expressed with *tub*-GAL4 with and without *DmManf* overexpression to study whether high levels of DmManf affected ubiquitous silencing of selected genes (see Additional file 4). B-C) DmManf (magenta) was ubiquitously expressed in the wing disc of 3rd instar larvae. MS1096-GAL4 expression detected by UAS-mCD8-GFP (green) was found mainly in the dorsal wing compartment but also in other regions of the wing disc (B). MS1096-GAL4 expression pattern was also detected in the CNS (C). Nuclear counterstain DAPI (gray) was used to mark the tissue morphology. Scale bar 50 μm. D) Insertion of GAL4 construct GawB in driver lines 69B-GAL4 and MS1096-GAL4 [31] did not affect adult fly phenotype (+/+, top row). Heterozygous *DmManf* mutation (*DmManf* ^*Δ96*^/+, middle row) or overexpression of *DmManf* (UAS-*DmManf* ^*L5*^, bottom row) together with 69B-GAL4 and MS1096-GAL4 insertions showed no obvious phenotype in adult flies. (TIFF 3486 kb)

Additional file 3: Examples of candidate genes for interacting partners of *DmManf*. A tiff file. A) MS1096-GAL4 was used to silence candidate genes in wild type, heterozygous *DmManf* ^*Δ96*^ mutant (*DmManf* ^*Δ96*^/+) and *DmManf* overexpression (+ *DmManf* OE) backgrounds. With many candidate genes, both heterozygous *DmManf* ^*Δ96*^ mutant background and *DmManf* overexpression resulted in more severe phenotype. For example, knockdown of *Tom20* (Translocase of outer membrane 20, a mitochondrial transmembrane transporter protein) showed a mildly wrinkled wing phenotype in wild type background (a1). When heterozygous *DmManf* ^*Δ96*^ mutant background (a2) or simultaneous *DmManf* overexpression was used (a3), wings were strongly wrinkled. Similarly, knockdown of *Cdk12* (a cyclin-dependent protein serine/threonine kinase; b1-b3) showed stronger phenotype both in heterozygous *DmManf* ^*Δ96*^ mutant and *DmManf*-overexpressing backgrounds. Complete list of the alterations is presented in Fig. 4. B) Quantitative analysis of ubiquitous knockdown of candidate genes *CSN3*, *Irbp*, *COQ7* and ubiquinone synthesis related gene *COQ2* showed altered pupal viability with *DmManf* overexpression (+ *DmManf* OE, red) in comparison to wild type background (− *DmManf* OE, blue). *tub*-GAL4/+ flies were used as wild type control. C) Proportion of emerged adults when candidate genes were ubiquitously knocked down with *tub*-GAL4 with (+ OE) or without (− OE) *DmManf* overexpression. *, *P* < 0.05; ***, *P* < 0.001, Student’s t-test. Amount of pupae and adults analysed in B-C are presented in Additional file 4. Proportion of Tb^+^ pupae was normalized to experimentally determined proportion of Tb^+^ pupae (see Additional file 8, wild type and wild type/SM6-TM6). (TIFF 1442 kb)

Additional file 4: Results from ubiquitous knockdown studies of UAS-RNAi lines with and without *DmManf* overexpression. A pdf file. (PDF 10 kb)

Additional file 6: Number of heterozygous pupae in the rescue experiments of *DmManf* ^*Δ96*^ mutant lethality by abolishment of *debcl*. A pdf file. (PDF 29 kb)

Additional file 7: List of UAS-RNAi lines used in the study. A pdf file. (PDF 5 kb)

Additional file 8: Results from ubiquitous knockdown of UAS-*DmManf*-RNAi. A pdf file. (PDF 18 kb)
