# Supplementary figures and images for: In vivo screening reveals interactions between Drosophila Manf and genes involved in the mitochondria and the ubiquinone synthesis pathway
Source: BMC Genet. 2017 Jun 2;18:52. doi: 10.1186/s12863-017-0509-3 (PMC5455201; doi:10.1186/s12863-017-0509-3)

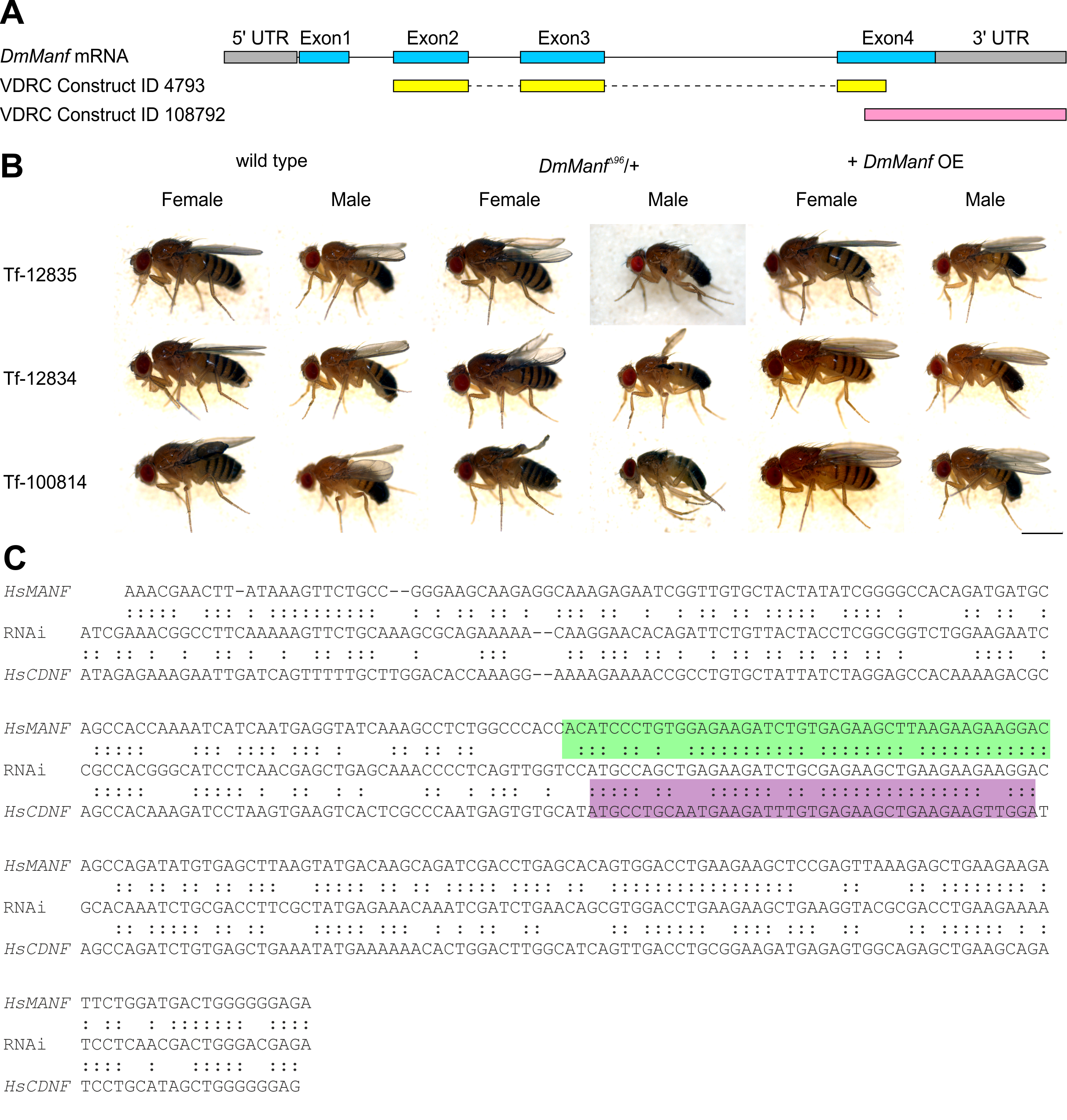

Supplement: Supplementary file 2 — Two DmManf-RNAi constructs in three independent transformant lines are available in VDRC. A tiff file. A) Alignment of DmManf-RNAi constructs 4793 and 108792. Construct 4793 targets last three exons of the DmManf gene with no predicted off-targets (VDRC data sheet). Construct 108792 targets exon 4 and 3′ UTR and has one predicted off-target (Sulfated/CG6725; VDRC data sheet). Construct 4793 in transformant line 12835 was used in further studies. B) Different UAS-DmManf-RNAi transformant lines show similar phenotypes when driven with wing-specific MS1096-GAL4. For all lines, heterozygous DmManf Δ96 mutant background lead to stronger wing phenotype and overexpression (OE) of DmManf rescued the wing phenotype. C) Alignment of UAS-DmManf-RNAi construct (ID 4793) with UAS-HsMANF and UAS-HsCDNF constructs. Strongest alignment is shown in green (HsMANF) and purple (HsCDNF). VDRC, Vienna Drosophila RNAi Center; Tf, transformant line; OE, overexpression. (TIFF 2536 kb) [file 12863_2017_509_MOESM1_ESM.tif]

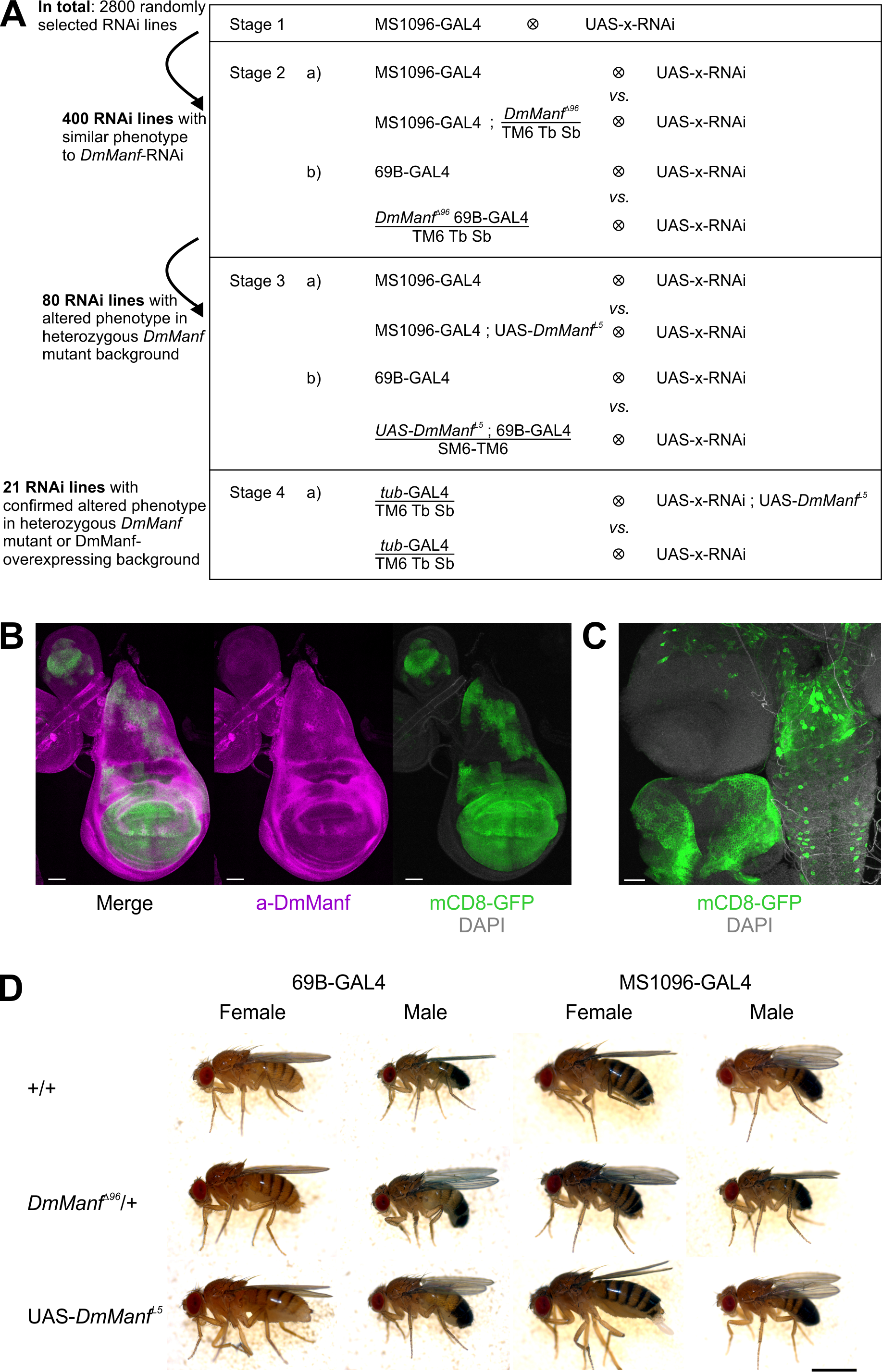

Supplement: Supplementary file 3 — Screen for genetic interaction partners of DmManf. A tiff file. A) Scheme of the crosses used in the partial RNAi library screen. First, randomly selected UAS-x-RNAi lines were crossed to wing-specific driver line MS1096-GAL4 (1). Lines showing similar phenotype to UAS-DmManf-RNAi lines were selected to the next stage. Second, two GAL4 drivers, wing-specific MS1096-GAL4 and semi-ubiquitous 69B-GAL4, were used in both wild type and heterozygous DmManf Δ96 mutant background (2) – UAS-x-RNAi lines showing distinct phenotypes in wild type and heterozygous DmManf Δ96 mutant backgrounds were selected. Secondary stage was repeated for selected UAS-x-RNAi lines in order to ensure the observed interactions. At stage 3, 69B-GAL4 and MS1096-GAL4 drivers were used to express UAS-x-RNAi lines with or without UAS-DmManf overexpression construct (3). Based on stages 2 and 3, 21 UAS-x-RNAi lines were selected as candidates for final stage 4. (4) UAS-x-RNAi lines were expressed with tub-GAL4 with and without DmManf overexpression to study whether high levels of DmManf affected ubiquitous silencing of selected genes (see Additional file 4). B-C) DmManf (magenta) was ubiquitously expressed in the wing disc of 3rd instar larvae. MS1096-GAL4 expression detected by UAS-mCD8-GFP (green) was found mainly in the dorsal wing compartment but also in other regions of the wing disc (B). MS1096-GAL4 expression pattern was also detected in the CNS (C). Nuclear counterstain DAPI (gray) was used to mark the tissue morphology. Scale bar 50 μm. D) Insertion of GAL4 construct GawB in driver lines 69B-GAL4 and MS1096-GAL4 [31] did not affect adult fly phenotype (+/+, top row). Heterozygous DmManf mutation (DmManf Δ96/+, middle row) or overexpression of DmManf (UAS-DmManf L5, bottom row) together with 69B-GAL4 and MS1096-GAL4 insertions showed no obvious phenotype in adult flies. (TIFF 3486 kb) [file 12863_2017_509_MOESM2_ESM.tif]

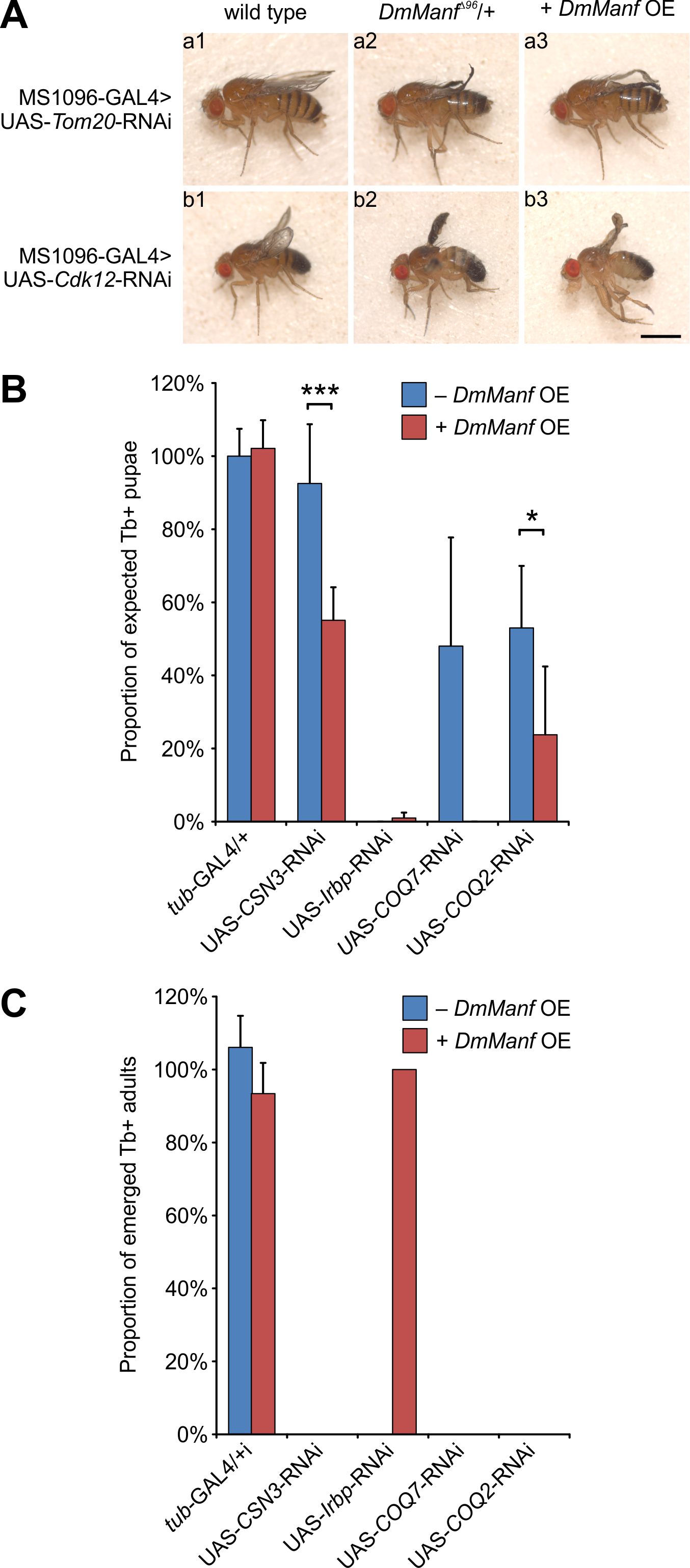

Supplement: Supplementary file 4 — Examples of candidate genes for interacting partners of DmManf. A tiff file. A) MS1096-GAL4 was used to silence candidate genes in wild type, heterozygous DmManf Δ96 mutant (DmManf Δ96/+) and DmManf overexpression (+ DmManf OE) backgrounds. With many candidate genes, both heterozygous DmManf Δ96 mutant background and DmManf overexpression resulted in more severe phenotype. For example, knockdown of Tom20 (Translocase of outer membrane 20, a mitochondrial transmembrane transporter protein) showed a mildly wrinkled wing phenotype in wild type background (a1). When heterozygous DmManf Δ96 mutant background (a2) or simultaneous DmManf overexpression was used (a3), wings were strongly wrinkled. Similarly, knockdown of Cdk12 (a cyclin-dependent protein serine/threonine kinase; b1-b3) showed stronger phenotype both in heterozygous DmManf Δ96 mutant and DmManf-overexpressing backgrounds. Complete list of the alterations is presented in Fig. 4. B) Quantitative analysis of ubiquitous knockdown of candidate genes CSN3, Irbp, COQ7 and ubiquinone synthesis related gene COQ2 showed altered pupal viability with DmManf overexpression (+ DmManf OE, red) in comparison to wild type background (− DmManf OE, blue). tub-GAL4/+ flies were used as wild type control. C) Proportion of emerged adults when candidate genes were ubiquitously knocked down with tub-GAL4 with (+ OE) or without (− OE) DmManf overexpression. *, P < 0.05; ***, P < 0.001, Student’s t-test. Amount of pupae and adults analysed in B-C are presented in Additional file 4. Proportion of Tb+ pupae was normalized to experimentally determined proportion of Tb+ pupae (see Additional file 8, wild type and wild type/SM6-TM6). (TIFF 1442 kb) [file 12863_2017_509_MOESM3_ESM.tif]
