# Supplementary material for: In vivo screening reveals interactions between Drosophila Manf and genes involved in the mitochondria and the ubiquinone synthesis pathway
Source: BMC Genet. 2017 Jun 2;18:52. doi: 10.1186/s12863-017-0509-3 (PMC5455201; doi:10.1186/s12863-017-0509-3)
Supplement: Supplementary file 5 — Results from ubiquitous knockdown studies of UAS-RNAi lines with and without DmManf overexpression. A pdf file. (PDF 10 kb) [file 12863_2017_509_MOESM4_ESM.pdf]

Additional file 4 Results from ubiquitous knockdown studies of UAS-RNAi lines with and without *DmManf* overexpression.

	Without *DmManf* OE				With *DmManf* OE			
**UAS-RNAi line**	**Tb+**	**Tb-**	**pupal-%**	**adult-%**	**Tb+**	**Tb-**	**pupal-%**	**adult-%**
UAS-*bora*-RNAi[1]	20	612	6%	0%	6	461	3%	0%
UAS-*betaTry*-RNAi	126	118	97%	109%	53	107	104%	98%
UAS-*dome*-RNAi	102	118	87%	103%	68	109	120%	75%
UAS-*Hsp68*-RNAi	1	166	1%	0%	0	139	0%	0%
UAS-*l (2) 37Bb*-RNAi[2]	0	91	0%	0%	0	80	0%	0%
UAS-*ND75*-RNAi	0	218	0%	0%	0	94	0%	0%
UAS-*Aats-phe*-RNAi	0	133	0%	0%	0	103	0%	0%
UAS-*pABp*-RNAi	0	76	0%	0%	0	116	0%	0%
UAS-*Spn27A*-RNAi	110	115	92%	1%	72	117	119%	1%
UAS-*Taf5*-RNAi	0	185	0%	0%	0	95	0%	0%
UAS-*timeout*-RNAi[1,2]	282	495	112%	0%	205	381	108%	0%
UAS-*Tom20*-RNAi[1,2]	1	376	1%	100%	1	340	1%	100%
UAS-*Ts*-RNAi	28	104	40%	0%	11	63	46%	0%
UAS-*Fip1*-RNAi	0	186	0%	0%	0	178	0%	0%
UAS-*Cdk12*-RNAi	0	172	0%	0%	0	203	0%	0%
UAS-*CG9249*-RNAi	0	127	0%	0%	0	128	0%	0%
UAS-*CG7277*-RNAi	151	153	94%	77%	82	164	104%	98%
UAS-*CG30493*-RNAi	1	115	2%	0%	1	70	4%	0%
UAS-*CG32174*-RNAi	113	79	111%	85%	49	89	111%	96%
UAS-*Irbp*-RNAi[1,2]	0	457	0%	0%	2	522	1%	100%
UAS-*COQ7*-RNAi[1]	153	376	48%	0%	0	226	0%	0%
UAS-*CG9613*-RNAi[1]	217	523	53%	0%	22	249	24%	0%
UAS-*CSN3*-RNAi[1]	285	304	93%	0%	107	487	55%	0%

tub-GAL4/TM6 Tb Sb females were crossed to UAS-*x*-RNAi(/SM6-TM6) (without *DmManf* OE) and UAS-*x*-RNAi ; UAS-*DmManf*-OE/SM6-TM6 (with *DmManf* OE) males. Columns: Tb^+ and Tb^-, amounts of Tb^+ and Tb^- pupae in crosses; pupal-%, normalized proportion of Tb^+ of all pupae, wild type or wild type/SM6-TM6 were used to normalize proportions (see Additional file 7); adult-%, proportion of emerged adults out of Tb^+ pupae. OE, overexpression. n of analysed vials = 2 (except [1], n = 6 vials, see Methods). Without *DmManf* overexpression, homozygous UAS-*x*-RNAi lines were used expect [2] which were balanced with SM6-TM6.
