# Supplementary material for: In vivo screening reveals interactions between Drosophila Manf and genes involved in the mitochondria and the ubiquinone synthesis pathway
Source: BMC Genet. 2017 Jun 2;18:52. doi: 10.1186/s12863-017-0509-3 (PMC5455201; doi:10.1186/s12863-017-0509-3)
Supplement: Supplementary file 6 — Number of heterozygous pupae in the rescue experiments of DmManf Δ96 mutant lethality by abolishment of debcl. A pdf file. (PDF 29 kb) [file 12863_2017_509_MOESM6_ESM.pdf]

Additional file 6 Number of heterozygous pupae in the rescue experiments of *DmManf*$^{\Delta 96}$ mutant lethality by abolishment of *debcl*.

Genotype	Type of *debcl* allele	Number of balanced pupae
debcl^{W105}/CyO; *DmManf*$^{\Delta 96}$/TM6 Tb Sb	dominant negative	195
debcl^{E26}/CyO; *DmManf*$^{\Delta 96}$/TM6 Tb Sb	loss of function	297
debcl-RNAi*; DmManf*$^{\Delta 96}$/SM6-TM6 (x) *da*-GAL4 *DmManf*$^{\Delta 96}$/TM6 Tb Sb	knockdown	135

Progeny of each genotype indicated was analysed. Tb^+ and Tb^- pupae were counted. No homozygous *DmManf*$^{\Delta 96}$ mutant pupae (Tb^+) were found in any of the rescue experiments listed here. Number of vials analysed = 2.
