# Supplementary material for: In vivo screening reveals interactions between Drosophila Manf and genes involved in the mitochondria and the ubiquinone synthesis pathway
Source: BMC Genet. 2017 Jun 2;18:52. doi: 10.1186/s12863-017-0509-3 (PMC5455201; doi:10.1186/s12863-017-0509-3)
Supplement: Supplementary file 7 — List of UAS-RNAi lines used in the study. A pdf file. (PDF 5 kb) [file 12863_2017_509_MOESM7_ESM.pdf]

Additional file 7: List of UAS-RNAi lines used in the study

Gene name	Symbol	CG	Tf ID	Library
aurora borealis	*bora*	CG6897	35134	GD
betaTrypsin	*betaTry*	CG18211	102898	KK
COP9 complex homolog subunit 3	*CSN3*	CG18332	12822	GD
COQ7	*COQ7*	CG14437	17602	GD
domeless	*dome*	CG14226	36356	GD
Heat shock protein 68	*Hsp68*	CG5436	5436R-1	NIG
Inverted repeat-binding protein	*Irbp*	CG5247	5247R-2	NIG
lethal (2) 37Bb	*l (2) 37Bb*	CG10655	10655R-1	NIG
NADH:ubiquinone reductase 75kD subunit precursor	*ND75*	CG2286	100733	KK
Phenylalanyl-tRNA synthetase	*Aats-phe*	CG13348	13348R-2	NIG
polyA-binding protein	*pABp*	CG5119	22007	GD
Serpin-27A	*Spn27A*	CG11331	107404	KK
TBP-associated factor 5	*Taf5*	CG7704	45957	GD
timeout	*timeout*	CG7855	7855R-1	NIG
Translocase of outer membrane 20	*Tom20*	CG7654	7654R-1	NIG
Ts	*Ts*	CG3181	3181R-1	NIG
-	*Fip1*	CG1078	27317	GD
-	*CG4707*	CG4707	4707R-1	NIG
-	*CG6455*	CG6455	47615	GD
-	*Cdk12*	CG7597	25508	GD
-	*CG9249*	CG9249	47643	GD
Coenzyme Q biosynthesis protein 2	*COQ2*	CG9613	108373	KK
-	*CG7277*	CG7277	30691	GD
-	*CG30493*	CG30493	105722	KK
-	*CG32174*	CG32174	100816	KK
death executioner Bcl-2 homologue	*debcl*	CG33134	47518	GD

Symbols used: CG, CG number of the gene (http://www.flybase.org); Tf ID, transformant identification; Library, RNAi library in which GD = Vienna *Drosophila* RNAi Center (VDRC) GD library, KK = VDRC KK library, NIG = National Institute of Genetics library.
