# Supplementary material for: In vivo screening reveals interactions between Drosophila Manf and genes involved in the mitochondria and the ubiquinone synthesis pathway
Source: BMC Genet. 2017 Jun 2;18:52. doi: 10.1186/s12863-017-0509-3 (PMC5455201; doi:10.1186/s12863-017-0509-3)
Supplement: Supplementary file 8 — Results from ubiquitous knockdown of UAS-DmManf-RNAi. A pdf file. (PDF 18 kb) [file 12863_2017_509_MOESM8_ESM.pdf]

Additional file 8 Results from ubiquitous knockdown of UAS-*DmManf*-RNAi.

UAS line	Tb^+	Tb^-	pupal-%	adult-%
wild type *	489	423	100%	106%
wild type/SM6-TM6 *	138	279	100%	106%
UAS-*DmManf*-RNAi	11	504	5%	0%
UAS-*DmManf*-RNAi ; UAS-*DmManf*-OE/SM6-TM6	148	293	103%	86%
UAS-*DmManf*-RNAi ; UAS-mCD8-GFP	11	467	5%	17%
UAS-*DmManf*-RNAi ; UAS-*HsMANF*-OE/SM6-TM6	193	402	100%	93%
UAS-*DmManf*-RNAi ; UAS-*HsCDNF*-OE/SM6-TM6	199	349	113%	82%
UAS-*DmManf*-RNAi ;$DmManf^{\Delta 96}$/SM6-TM6	0	304	0%	0%
UAS-*DmManf*-OE *	444	359	102%	93%

tub-GAL4/TM6 Tb Sb females were crossed to males of indicated phenotypes. Columns: Tb^+ and Tb^-, amounts of Tb^+ and Tb^- pupae in crosses; pupal-%, normalized proportion of Tb^+ of all pupae, wild type or wild type/SM6-TM6 were used to normalize proportions; adult-%, proportion of emerged adults out of Tb^+ pupae. OE, overexpression. n of analysed vials = 6. * Wild type and UAS-*DmManf*-OE data has been previously reported in [13].
